# Epigenomic analysis identifies DTP subpopulation using HOPX to develop targeted therapy resistance in lung adenocarcinoma

**DOI:** 10.1016/j.isci.2025.112387

**Published:** 2025-04-13

**Authors:** Yang Tian, Reshmee Bhattacharya, Seungyeul Yoo, Feng Jiang, Eric Park, Genesis Lara Granados, Yudao Shen, Kwang-Su Park, Husnu Umit Kaniskan, Jian Jin, Benjamin D. Hopkins, Jun Zhu, Hideo Watanabe

**Affiliations:** 1 Division of Pulmonary, Critical Care and Sleep Medicine, Department of Medicine, Icahn School of Medicine at Mount Sinai, New York, NY, USA; 2 Tisch Cancer Institute, Icahn School of Medicine at Mount Sinai, New York, NY, USA; 3 Department of Genetics and Genomic Sciences, Icahn School of Medicine at Mount Sinai, New York, NY, USA; 4 Icahn Institute for Data Science and Genomic Technology, New York, NY, USA; 5 GeneDx, Stamford, CT, USA; 6 Mount Sinai Center for Therapeutics Discovery, Departments of Pharmacological Science, Oncological Science and Neuroscience, Icahn School of Medicine at Mount Sinai, New York, NY, USA; 7 Englander Institute for Precision Medicine, Weill Cornell Medicine, New York, NY, USA; 8 Department of Physiology and Biophysics, Weill Cornell Medicine, New York, NY, USA

**Keywords:** Therapeutics, Epigenetics, Integrative aspects of cell biology, Cancer, Transcriptomics

## Abstract

Genomic studies have identified oncogenic drivers in lung cancer, enabling effective targeted therapies. However, patients who initially respond inevitably experience regrowth. The drug-tolerant persister (DTP) stage is a key source of non-genetic resistance, yet its epigenetic regulation remains unclear. Using single-cell chromatin accessibility profiling (scATAC-seq), we identified two distinct DTP subpopulations in EGFR- and KRAS-inhibited models. The integrative network and pathway analysis revealed that one subpopulation is associated with cell cycle, while the other is enriched in developmental pathways. HOPX was the most enriched alveolar signature gene in the latter. It was transiently upregulated with cytoplasmic-to-nuclear translocation, and its deletion significantly delayed DTP regrowth. Mechanistically, HOPX regulates NF-κB activation and repressive histone modifications. Combining targeted therapy with NF-κB or histone-methyltransferase inhibitors nearly abolished DTP regrowth. These findings highlight a potential anti-relapse strategy by targeting developmental pathways to modulate key lineage factors during lung regeneration in patients relapsing on targeted therapy.

## Highlights

• scATAC-seq reveals two distinct DTP subpopulations in lung cancer
• HOPX is upregulated in one DTP subpopulation and its loss delays regrowth
• HOPX regulates NF-κB and repressive histone modifications in therapy resistance

## Introduction

Lung cancer is the leading cause of cancer-related mortality, and lung adenocarcinoma (LUAD) accounts for approximately 40% of the cases.^1^ Comprehensive genome profiling of non-small cell lung cancer (NSCLC) has identified key driver gene alterations mainly in LUAD, such as *EGFR*, *KRAS*, and *ALK.*^2^ Inhibitors targeting these driver mutations have significantly improved the patients’ outcomes. However, patients who initially respond to targeted therapy inevitably experience disease progression often within a year of the treatment.^3^ For example, 48% of patients who initially responded to osimertinib, a third-generation EGFR-TKI that targets both sensitizing and gatekeeper T790M mutations of EGFR, progressed within median follow-up time of 15 months.^4^ Besides the genetic contribution to acquired resistance either through selection of pre-existing mutations or emergence of new somatic genetic alterations upon drug treatment, increasing evidence suggests crucial roles of non-genetic mechanisms during the evolution of drug resistance.^5^ Epigenetic alterations such as DNA and histone modifications, which can lead to transcriptomic changes through affecting transcription factor binding, have been shown to drive resistance to targeted therapies.^5^^,^^6^ For example, a histone modifying enzyme KDM5A, which primarily mediates histone H3K4 demethylation, has been reported to be elevated after targeted therapy and plays a role in resistance development.^7^ In contrast to the extensively modeled genetic mechanisms identified through genome-scale sequencing approaches, much less is known about the epigenomic heterogeneity and dynamics during the evolution of drug resistance. Therefore, identifying underlying epigenetic mechanisms is a critical step forward to develop therapeutic strategies to suppress emerging drug resistance effectively.

Recent studies identified a reversible and transient stage of cancer cells entering a quiescent state to ensure survival before adapting to the rewired signaling activity and re-entering cell cycle, defined as drug-tolerant persister (DTP) cells, which can be clinically observed as residual disease, potentially a major source of recurrence.^7^^,^^8^ The evolution of drug resistance requires cellular plasticity that involves reprogramming to acquire drug-refractory phenotypes^9^; for example, the positive role of epithelial–mesenchymal transition (EMT) in developing acquired drug resistance has been demonstrated in many cancer models.^10^ Another type of cellular plasticity involved in drug resistance is lineage plasticity, which represents a process whereby cancer cells transit from one cell lineage to another. In particular, neuroendocrine differentiation has been reported as one of major resistance mechanisms in prostate cancer^11^ and NSCLC.^12^^,^^13^ However, whether there are additional forms of plasticity regulating the evolution of drug resistance remains unknown. Malignant cells have been shown to hijack developmental processes involved in reprogramming to acquire plasticity.^14^ In autochthonous LUAD mouse models, the precursor adenomas typically have high expression of alveolar markers which are gradually lost during the progression to LUAD.^15^ Coincidently, a recent study analyzing scRNA-seq data from lung cancer biopsies from patients with residual disease (representing a DTP state) showed an elevated alveolar gene signature.^16^ The presence of more alveolar-like phenotype in both tumor initiation and drug-induced resistance suggests the connection between these two processes, i.e., the DTP stage can be viewed as a tumor re-initiation where it allows future tumor development through modulating lung developmental pathways. One supporting evidence is from a study that showed YAP/TEAD activity was increased in DTP cells,^17^ while increasing evidence suggests Hippo/Yap plays important roles not only in lung progenitor cell differentiation^18^ but also in lung tumorigenesis.^19^ The positive correlation of Hippo/Yap on drug resistance process and tumor initiation were also widely demonstrated in other types of cancers.^20^

Given the reversible nature of DTP stage, which also requires reprograming plasticity, epigenomic regulation is considered one of the major mechanisms essential for this phenotype. In a lung cancer model undergoing EGFR-tyrosine kinase inhibitor (TKI) therapeutics, a main feature of the DTP stage is a global repressive chromatin state characterized by increased repressive histone H3 modifications.^21^ Treatment with histone deacetylase (HDAC) inhibitors, which leads to a less compacted chromatin state, suppresses the regrowth of DTP,^21^ indicating the importance of heterochromatin in maintaining the DTP state. However, another study in breast cancer reported that H3K27me3 conditions chemo-tolerance, where simultaneous demethylase inhibition and chemotherapy treatment delays tumor recurrence.^22^ The apparent contradictory evidence suggests that the effect of heterochromatin on drug tolerance is different depending on the context, i.e., tumor types and treatment, which may be dynamic and heterogeneous even under the same context.

Emerging single cell sequencing techniques provide a way to resolve the heterogeneity and plasticity at multiple stages of tumor development at a transcription level (scRNA-seq) and an epigenetic level (single-cell chromatin accessibility profiling, scATAC-seq, transposase-accessible chromatin).^23^ While scATAC-seq yields a less dynamic range than scRNA-seq, it provides insights into regulatory elements and better captures cell lineage identity.^24^ In this study, we depicted the lineage heterogeneity of DTP stage based on chromatin accessibility, and identified that a key lung development factor HOPX is transiently upregulated at an early phase of DTP and is essential for DTP development. Further mechanistic studies showed that HOPX regulates NF-κB activation and repressive histone modifications, which are essential for DTP survival and regrowth.

## Results

### Single cell ATAC sequencing (scATAC-seq) reveals heterogeneous subpopulations of osimertinib-induced DTP stage

**Figure 1:**
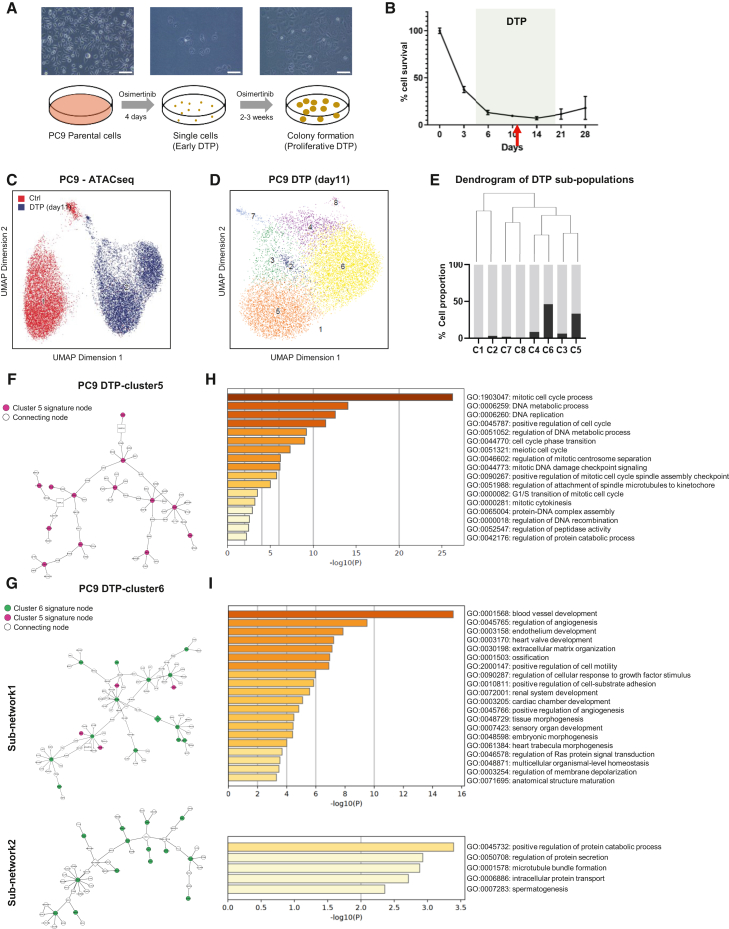
The heterogeneity of osimertinib-induced DTP stage revealed by scATAC-seq (A) Representative bright-field microscopic images (Scale bar 100μm) and schematic diagram of PC9 DTP development under 150nM osimertinib treatment at different stages. (B) PC9 cells were treated with 150nM osimertinib starting at day 0 when confluency reached 100%, and cell viability was measured at indicated time points and normalized to day 0. The time frame of DTP is indicated between dashed lines, and the red arrow indicates the time point (day 11) when scATAC-seq was performed. Data are presented as means ± S.D. (C and D) Heterogeneity of DTP cells (150nM, day 11) revealed by UMAP representation of scATAC-seq data. (C) Cells colored by sample; control (red) and osimertinib-induced DTP (blue). (D): Cells colored by unsupervised clusters of osimertinib-treated samples. (E) *Top*: Dendrogram of DTP subpopulations, *Bottom*: cell proportion of indicated cluster (black) and all the rest clusters (gray). (F and G) Subnetwork with gene nodes and connections at one layer when cluster 5 (F) or cluster 6 (G) signature (Table S3) is projected to LUAD Bayesian network. Pink nodes represent cluster 5 signature genes, and green nodes represent cluster 6 signature genes. Nodes indicated in square are key drivers of the subnetwork (see Methods). All nodes (genes) of subnetworks were listed in Table S4. (H and I) Gene ontology (GO) analysis of sub-networks nodes (genes) of cluster 5 and cluster 6 (Table S4).

To explore epigenomic features of targeted therapeutics-induced DTP stages in lung cancer, we first recapitulated an *in vitro* system with a commonly used PC9 cell model, which harbors an *EGFR* exon 19 deletion, undergoing treatment with 150nM osimertinib to induce a DTP stage. In this PC9 model, the cells continued to undergo cell death until day 4, from which point the cell viability was stable at 5–10% and those that survived stopped proliferating (Figures 1A and 1B). This was accompanied by a change to a rounder morphology, indicating that those cells entered into a DTP phase (Figure 1A). Two to three weeks after the initiation of the treatment, a proportion of the survived cells started dividing and forming colonies (Figures 1A and 1B), indicating their potential for regrowth as previously reported.^25^ To investigate the potential heterogeneity of epigenomic states in DTP cells, we performed scATAC-seq on untreated cells and osimertinib-induced DTP cells at day 11, a nadir time point after entering DTP and before the regrowth (Figure 1B). Unsupervised hierarchical clustering analysis and dimension reduction visualization with uniform manifold approximation and projection (UMAP) revealed that the untreated group has one major population (red) distinct from the cells at the DTP stage, while the osimertinib-induced DTP cells have two distinct subpopulations (blue) (Figure 1C), suggesting an increased heterogeneity of drug induced DTP stage. By using various thresholds for clustering, DTP cells exhibit consistently higher number of clusters. To understand the nature of epigenomic heterogeneity of DTP cells in detail, we re-clustered only cells at the DTP stage, dividing them into 8 sub-populations with a standard *Seurat* clustering resolution at 0.8 (Figure 1D). The heterogeneity of DTP stage was further confirmed at transcriptomic level by scRNA-seq in the same model (Figures S1A and S1B). From the hierarchical cluster structure of those subpopulations, we observed clusters 5 and 6 to be the two major sub-populations with a substantial distance between them (Figure 1E). Therefore, we focused on these two subpopulations to investigate the molecular features with epigenomic heterogeneity of DTP cells.

By marker gene analysis using accessibility surrounding the genes as a proxy to their transcriptional activity between clusters 5 and 6, we derived 353 cluster-5 signature genes and 552 cluster-6 signature genes (Figure S1C). To characterize their molecular function in the context of a specific biological network in LUAD, we projected those signature genes to a Bayesian regulatory network we have previously established, which consists of 8,533 genes constructed by integrating gene expression, CNV, and methylation information of LUAD.^26^ The integrative network analysis identified that cluster-5 signature forms one major sub-network with 63 nodes (genes), while cluster-6 signature consists of two large sub-networks with 85 and 53 nodes respectively (Figures 1F and 1G). The network nodes and their neighboring nodes within the first layer of regulations (edges) were used for GO pathway enrichment analysis. The results showed cluster-5 signature is enriched for cell proliferation functions such as mitotic process and DNA replication process (Figure 1F). In contrast, the largest sub-network of cluster-6 signature is related to developmental processes, and the second large sub-network is enriched for protein transport and secretion (Figure 1G). Taken together, these results suggest that DTP stage is more heterogeneous, consisting of two major sub-populations, which may be responsible for different biological functions in the process of drug tolerance.

### The two distinct DTP sub-populations are identified in KRAS-inhibitor induced DTP models

**Figure 2:**
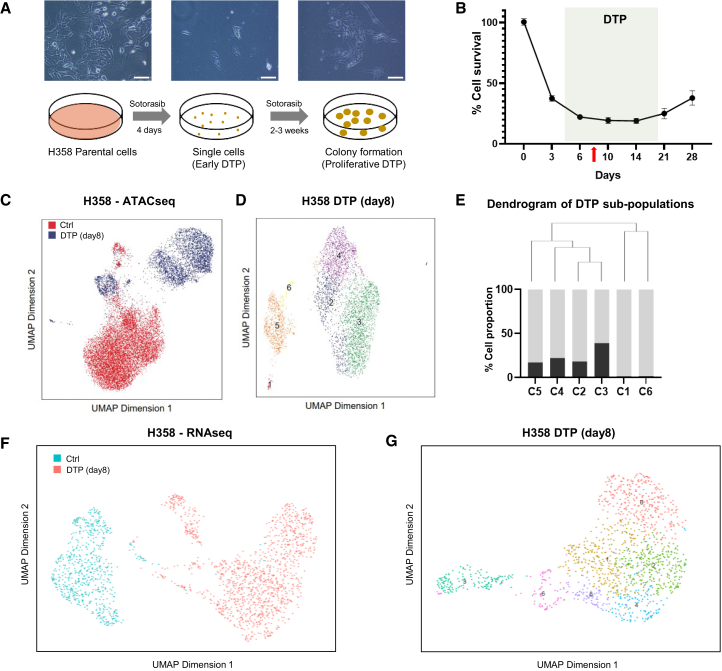
The heterogeneity of sotorasib-induced DTP stage revealed by scATAC-seq (A) Representative bright-field microscopic images (Scale bar 100μm) and schematic diagram of NCI-H358 DTP development under 500nM sotorasib treatment at different stages. (B) NCI-H358 cells were treated with 500nM sotorasib starting at day 0 when confluency reached 100%. Cell viability was measured at indicated time points and normalized to day 0. The time frame of DTP is indicated between dashed lines, and the red arrow indicates the time point (day 8) when scATAC-seq was performed. Data are presented as means ± S.D. (C and D). Heterogeneity of DTP cells (500nM, day 8) revealed by UMAP representation of scATAC-seq data. (C) Cells colored by sample; control (red) and sotorasib-treated DTP (blue). (D) Cells colored by unsupervised clusters of sotorasib-treated samples. (E) *Top*: Dendrogram of DTP subpopulations, *Bottom*: cell proportion of indicated cluster (black) and all the rest clusters (gray). (F and G) Heterogeneity of DTP cells (500nM, day 8) revealed by UMAP representation of scRNA-seq data. (F) Cells colored by sample, control (green) and sotorasib-treated (orange). (G) Cells colored by unsupervised clusters of sotorasib-treated samples.

Next, we sought to examine whether the increased heterogeneity and those two major epigenomic clusters are a general phenomenon in DTP cells during targeted-therapeutics induced resistance evolution. We established an independent *in vitro* model using KRAS-mutant NCI-H358 cells undergoing treatment with sotorasib (also known as AMG510), a KRAS G12C inhibitor. At 500 nM, the growth curve of NCI-H358 cells was similar to that in the PC9 model, where approximately 20% of cells survived and entered a DTP stage after 5 days of treatment (Figures 2A and 2B). The heterogeneity on chromatin accessibility of untreated cells and sotorasib-induced DTP cells (8 days after the initiation of the treatment) was assessed by scATAC-seq. The day 8 time point was chosen based on the defined DTP period, ensuring collection after DTP entry but before regrowth, as well as the observation that NCI-H358 cells exhibited a higher survival rate under sotorasib treatment compared to PC9 model (Figure 2B). Similar to the PC9 model, we observed two major sub-populations in the DTP cells (blue) (Figure 2C). The DTP cells induced by sotorasib were divided into 6 different sub-populations by unsupervised hierarchical clustering with the same parameters (Figure 2D), among which cluster-3 and cluster-4 were the two major sub-populations representing the distinct clusters by the dendrogram tree structure (Figure 2E). Further, scRNA-seq data confirmed the heterogeneity of DTP stage, where the untreated NCI-H358 cell has one main population while sotorasib-induced DTP cells has two major sub-populations (Figure 2F). Unsupervised clustering divided this DTP stage to 7 sub-populations, similar to the classification based on scATAC-seq analysis (Figure 2G).

Marker gene analysis between clusters 3 and cluster 4 revealed 777 cluster-3 signature genes and 1,076 cluster-4 signature genes (Figure S2A). Again, the signature genes were further projected to the same Bayesian network as in the PC9 model. The results showed cluster 3 consists of three sub-networks and cluster 4 with two sub-networks (Figures S2B and S2C). Pathway enrichment analysis of those sub-networks indicate that cluster 3 cells were functionally associated with cell motility, catabolic processes, and wound healing, while cluster 4 cells were associated with immune activation and chromosome disorganization (Figures S2B and S2C). Sotorasib-induced DTP subpopulations represent more diverse and distinct functions involved in basic cellular processes compared to PC9 model, while processes involving nucleic acid metabolism and cell division are shared between the two models. This suggests DTP state is not governed by a singular mechanism but diverse mechanisms contribute to survival and resistance under drug stress. Nonetheless, these results demonstrated the epigenomic heterogeneity of DTP stage in different cell models induced by different targeted therapies, and we sought to investigate whether the two major sub-populations that arise may represent a common feature of targeted-therapeutics-induced DTP in lung cancer.

### One DTP subpopulation is consistently marked by alveolar signature gene HOPX

**Figure 3:**
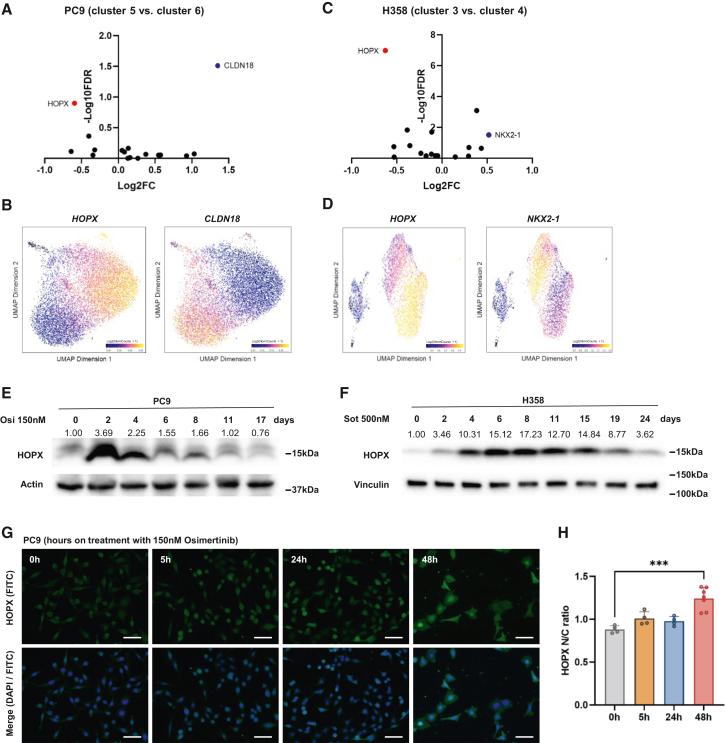
HOPX is specifically enriched in one DTP subpopulation and is transiently upregulated in DTP cells (A) Volcano plot showing differential accessibility at 17 established alveolar signature genes between PC9-DTP clusters 5 and 6. (B) UMAP of scATAC-seq data showing accessibility at *HOPX* and *CLDN18* gene loci. (C) Volcano plot showing differential accessibility at 17 established alveolar signature genes between NCI-H358-DTP cluster 3 and cluster 4. (D) UMAP of scATAC-seq data showing accessibility at *HOPX* and *NKX2-1* gene loci. (E) HOPX level measured by western blot on PC9 cells at 0, 2, 4, 6, 8, 11, and 17 days post initiation of 150nM osimertinib treatment. (F) HOPX level measured by western blot on NCI-H358 cells at 0, 2, 4, 6, 8, 11, 15, 19, and 24 days post initiation of 500nM sotorasib treatment. Quantification of the blot was measured by bands intensity and normalized to Actin or Vinculin and the untreated control group, and was labeled above the blot. Three independent experiments were conducted. (G) Immunofluorescent staining of HOPX in PC9 cells at indicated time points upon 150nM osimertinib treatment. Green: FITC, HOPX, Blue: DAPI, nuclei. Scale bar 100μm. (H) The cytoplasmic versus nucleus localized HOPX in immunofluorescent images were quantified by ImageJ software. HOPX N/C ratio was calculated by the mean nuclear intensity divided by the mean cytoplasmic intensity of FITC channel. Data are presented as means ± S.D. (*n* = 4). Statistical significance was determined using Kruskal-Wallis-Test, ∗∗∗*p* < 0.001.

Given the signature enrichment of developmental pathways in one of the major subpopulations under DTP, we speculated that the evolution of drug resistance potentially shares similarities with organ regeneration. Both require terminally differentiated cells to regain cellular plasticity, which serve as progenitors to re-populate differentiated cells. Given the recent evidence showing presence of alveolar gene signature enrichment in both tumor initiation^15^ and drug-induced resistance evolution,^16^ we hypothesized that lineage factors essential for lung development contribute to the early DTP stage. Upon evaluating the differential accessibility of 17 established alveolar signature genes,^27^^,^^28^ we found that the members of alveolar signature genes showed different distribution patterns among DTP sub-populations (Figures S3A and S3B). By comparing the two major DTP sub-populations, *HOPX* and *CLDN18* were respectively identified as the most differential alveolar marker in those sub-populations in the PC9 DTP model (Figures 3A and 3B). In the NCI-H358 DTP model, the most significantly differential alveolar markers for each subpopulation was *HOPX* and *NKX2-1*, respectively (Figures 3C and 3D). Similar HOPX expression pattern was also observed at transcriptional level where one DTP sub-population highly expresses *HOPX*, while the other sub-population enriched for *NKX2-1* revealed by scRNA-seq analysis (Figure S3C). The consistent enrichment of *HOPX* in a sub-population in two DTP models indicates a potentially universal role of HOPX regulating DTP composition and/or development. In addition, we assessed overlaps of the all marker genes of HOPX(+) DTP sub-populations between PC9 model (cluster 6) and NCI-H358 model (cluster 3) as well as HOPX(−) DTP sub-populations (cluster 5 in PC9 and cluster 4 in NCI-H358) (Figure S3D). Hypergeometric analysis showed a significant marker gene overlap between HOPX(+) PC9-DTP cells and HOPX(+) NCI-H358-DTP cells (Figure S3E), suggesting a common feature of HOPX(+) sub-populations in acquired drug resistance process.

The homeodomain-only protein homeobox (HOPX) is required for the plasticity of AT1 cells to dedifferentiate into AT2 cells during lung regeneration, indicating a progenitor cell like potential of HOPX (+) cells.^29^ Besides lung development, HOPX is also essential during cardiac development,^30^^,^^31^ consistent with our observation that HOPX(+) DTP sub-population (cluster 6 in PC9 model) was also enriched for cardiac development pathways (Figure 1I). Therefore, we further pursued to evaluate functional significance of HOPX on the development of DTP.

## HOPX is transiently upregulated in DTP cells and plays a role in DTP development

To further confirm the association of HOPX in DTP, we first determined the dynamics of HOPX expression during the DTP development. By measuring *HOPX* mRNA level by qRT-PCR at multiple time points during the DTP stage, we found the *HOPX* expression is induced around day 4, remains at a relative high level for 2 weeks, and decreases to the baseline level after the emergence of the re-grown population (Figures S3F and S3G), which indicate a potential functional role of HOPX for the early phase of DTP stage. Other alveolar lineage genes including *CLND18* and *NKX2-1* were also transiently upregulated, while another alveolar type I cell marker *AGER* remained unchanged (Figures S3F and S3G). These results were further confirmed at protein level by western blots where cells that underwent the osimertinib treatment showed HOPX upregulation as early as day 2 (Figure 3E). A similar transient induction of HOPX was observed in the sotorasib-induced NCI-H358 DTP model as well (Figure 3F). To visualize temporal and localization evolutionary dynamics of HOPX expression at single cell resolution, we also performed immunofluorescence assay and found HOPX expression was mainly in cytoplasm before osimertinib treatment and translocated to nuclei 48h after drug treatment (Figures 3G and 3H). Given the main function of HOPX as a transcriptional regulator, this nuclear localization further supports our hypothesis that HOPX exerts its function at the early phase of DTP.

### HOPX deletion diminishes the progress of DTP regrowth

**Figure 4:**
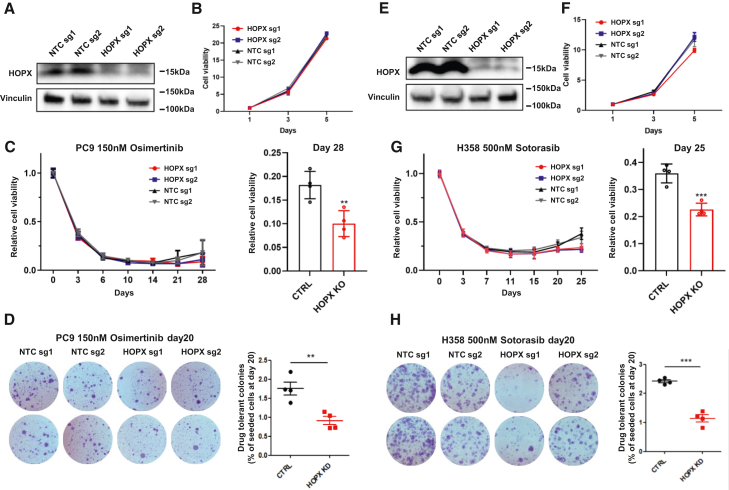
Deletion of HOPX impairs the progress of DTP regrowth (A) Western blot showing reduction of HOPX expression in PC9 cells by CRISPR-Cas9 mediated deletion of *HOPX* gene. (B) Cell growth curve of PC9 cells transfected with sgRNAs targeting *HOPX* or non-target sgRNAs. (C) Cell viability of PC9 cells transfected with sgRNAs targeting *HOPX* or non-target sgRNAs under the treatment of 150nM osimertinib at indicated time points (normalized to day0). *Right:* Bar graph showing cell viability at day 28 (*n* = 4, including duplicates for sgRNA1 and duplicates for sgRNA2). (D) Colony formation assay on PC9 cells, at seeding density of 150,000 cells/well under 150nM osimertinib starting at 24 h after seeding. *Left:* Two representative images taken at 20 days after treatment per condition. *Right:* Quantification of colony numbers based on the representative fields shown on the left (*n* = 4), including duplicates for sgRNA1 and duplicates for sgRNA2. (E) Western blot showing reduction of HOPX expression in NCI-H358 cells. (F) Cell growth of NCI-H358 cells transfected with sgRNAs targeting *HOPX* or non-target sgRNAs. (G) Cell viability of NCI-H358 cells transfected with sgRNAs targeting *HOPX* or non-target sgRNAs under the treatment of 500nM sotorasib at indicated time points (normalized to day 0). *Right:* Bar graph showing cell viability at day 25 (*n* = 4, including duplicates for sgRNA1 and duplicates for sgRNA2). (H) Colony formation assay on NCI-H358 cells, at seeding density of 150,000 cells/well under 500nM sotorasib starting at 24 h after seeding. *Left:* Two representative images taken at 20 days after treatment per condition. *Right:* Quantification of colony numbers based on the representative fields shown on the left (*n* = 4), including duplicates for sgRNA1 and duplicates for sgRNA2. Data are presented as means ± S.D. (*n* = 4). Statistical significance was determined using Mann-Whitney test. ∗*p* < 0.05, ∗∗*p* < 0.01, ∗∗∗*p* < 0.001.

Next, to investigate the functional role of HOPX in DTP stage, we first deleted *HOPX* using CRISPR-Cas9 with two sgRNAs and confirmed effective reduction of its expression by Western blot (Figure 4A). The cell growth of *HOPX*-deleted cells and cells with non-target sgRNAs exhibited a similar basal growth rate without osimertinib treatment (Figure 4B). Under osimertinib treatment, *HOPX*-deleted cells exhibited a reduced regrowth detected at day 28 and onward compared to control cells (Figure 4C), suggesting a lesser regrowth potential of the HOPX-deficient DTP cells. *HOPX* deletion also resulted in significantly smaller number of colonies formed at day 20 under Osimertinib compared to PC9 cells with non-target sgRNAs (Figure 4D), further demonstrating the essential function of HOPX during DTP development. Similarly, CRISPR-Cas9-mediated *HOPX* deletion in NCI-H358 cells did not affect their basal growth rate (Figures 4E and 4F), while significantly delayed the regrowth of DTP cells under the treatment with sotorasib (Figures 4G and 4H). Overall, those data suggest that HOPX plays an important role during DTP development, while under the normal condition it is not essential for the growth of cancer cells.

### HOPX regulation converges with NF-κB pathway during DTP development

**Figure 5:**
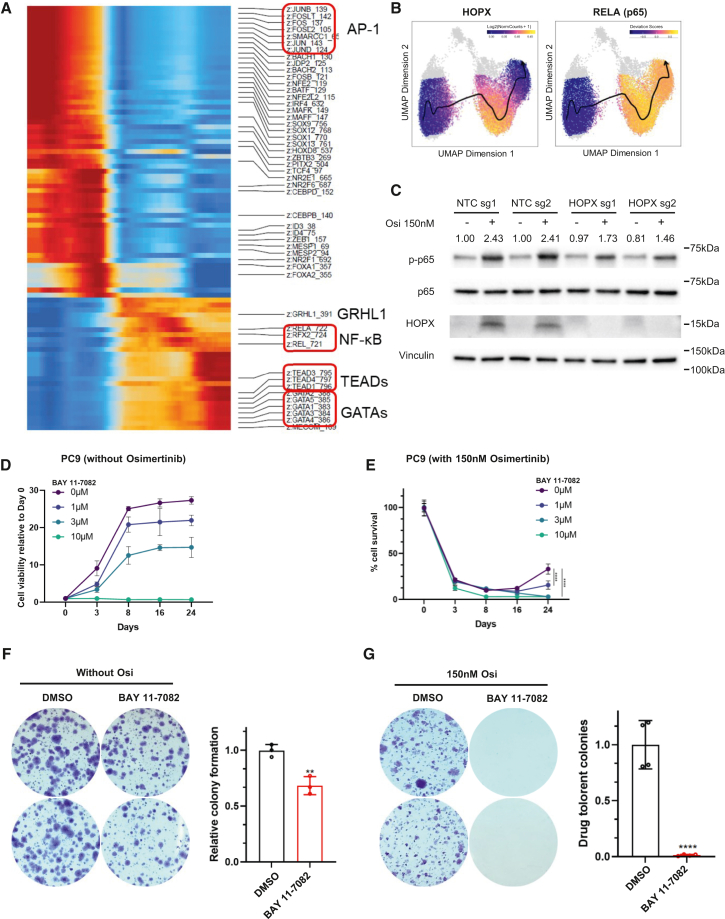
HOPX regulation converging with NF-κB pathway during DTP development (A) Heatmap showing enrichment of 86 differentially enriched transcription factor binding motif over the pseudotime trajectory from scATAC-seq constructed by *ArchR* based on gene accessibility. The horizontal axis represents individual single cells analyzed through scATAC-seq. Each column corresponds to the chromatin accessibility profile of a single cell, encompassing both untreated cells and DTP cells. The cells are ordered along the pseudotime trajectory, progressing from untreated cells to DTP cells, reflecting the transition in chromatin accessibility states during this process. (B) Pseudotime trajectory from scATAC-seq and projected *HOPX* and *RELA* gene accessibility on UMAP. Arrow represents directions of DTP cell state changes. (C) Western blot of p-p65, p65 and HOPX level in PC9 cells transduced with non-target sgRNAs or HOPX sgRNAs with or without osimertinib treatment. p65 phosphorylation level was quantified by bands intensity (phosphorylated/total), and normalized to vinculin and the untreated control group, and was labeled above the blot. (D) Cell growth of PC9 cells under different dosage of NF-κB inhibitor (BAY-11-7082) at indicated time points (normalized to day0). (E) Cell viability of PC9 cells under different dosage of BAY-11-7082 and 150nM osimertinib at indicated time points (normalized to day0). (F) Colony formation assay on PC9 cells, at seeding density of 1,000 cells/well, with or without 3μM BAY-11-7082 starting on the second day of seeding. Two representative images were taken on 14 days after treatment per condition (*left*). Quantification of colony numbers (*right*). (G) Colony formation assay on PC9 cells, at seeding density of 150,000 cells/well under 150nm osimertinib with or without 3μM BAY-11-7082 starting on the second day of seeding. Two representative images were taken on 20 days after treatment per condition (*left*). Quantification of colony numbers (*right*). Data are presented as means ± S.D. For (D) and (E), comparisons between indicated groups were analyzed using two-way ANOVA followed by Tukey’s tests (*n* = 8). For (F) and (G), Statistical significance was determined using Mann-Whitney test. *n* = 3 in (F) and *n* = 4 in (G). ∗*p* < 0.05, ∗∗*p* < 0.01, ∗∗∗*p* < 0.001, ∗∗∗∗*p* < 0.0001.

To investigate the mechanism as to how HOPX influences DTP development, we inferred a pseudotime trajectory based on the chromatin accessibility signals, which suggested that the shifting direction goes from untreated to HOPX+ DTP to HOPX- DTP (Figure S4A). By analyzing the difference between those accessible regions, we found that DNA-binding motifs for activator protein 1 (AP-1) components were significantly decreased after the drug treatment (Figures 5A and S4B). Since the AP-1 is known as one of the main downstream effectors of MAPK/ERK signaling, a major downstream of EGFR signaling pathway, the data likely captured osimertinib treatment causing a decrease in AP-1 activity. The motifs that were significantly enriched in DTP populations included Grainyhead and NF-κB motifs in the HOPX+ subpopulation, and TEADs and GATAs in the HOPX-subpopulation along the trajectory. (Figures 5A and S4B). The YAP/TEADs pathways have been widely reported to serve a positive role in acquiring drug resistance.^17^ We tested the expression of YAP/TAZ during DTP development and found that YAP initially decreased but later increased at the same time point when the regrowth of DTP started, while TAZ was continuously increased during DTP development (Figure S4C). The dynamic changes in YAP expression, along with increased chromatin accessibility during DTP, suggest that YAP may play a functional role in this process, consistent with previous studies highlighting the involvement of YAP/TEADs pathways in drug resistance. The NF-κB pathway was reported to be acutely activated in response to EGFR inhibition and contributes to cell survival.^32^ We evaluated the activity of NF-κB at different time points during the drug treatment, finding that an immediate decrease in NF-κB activity upon initiation of the osimertinib treatment followed by a significant increase in its activity (Figure S4D). While the motif accessibility of AP-1, NF-κB, and TEADs significantly changed after treatment, we did not observe a significant difference at their expression level (Figure S4E). These results suggest that increased functional activity of TEADs and NK-κB in DTP stage is likely contributed by more permissive chromatin structure of their targets and/or their post-translational modification by signaling activation, but not by their expression level.

When we investigated the difference between the two DTP subpopulations, we noticed NF-κB motif enrichment is specifically more prominent in HOPX+ DTP sub-populations along the pseudotime trajectory (Figures 5A and 5B). This prompted us to evaluate if there is a connection between HOPX regulation and NF-κB pathway during the DTP development. To determine whether HOPX influences NF-κB pathway activity in this context, we evaluated phosphorylation status of NF-κB upon osimertinib treatment in *HOPX*-deleted PC9 cells. The results showed that *HOPX*-deleted cells exhibited a decreased NF-κB activity compared to control cells under osimertinib treatment (Figure 5C). Furthermore, we found an NF-κB pathway inhibitor BAY11-7082 significantly, but only moderately, inhibits the cell growth of PC9 cells in a dosage dependent manner, indicating some degree of contribution from NF-κB pathway activity under the untreated condition for their survival (Figure 5D). Under the DTP condition, however, co-treatment with osimertinib and 3μM of BAY11-7082 appeared to enhance the effect of osimertinib, resulting in a substantial reduction in DTP regrowth (Figure 5E). Colony formation assay verified this combinatorial treatment effect with osimertinib and 3μM BAY11-7082, where treatment with the NF-κB inhibitor resulted in ∼30% reduction on colony formation compared to the control DMSO group, while co-treatment with osimertinib and the NF-κB inhibitor almost completely inhibited colony formation (Figures 5F and 5G). Overall, these results suggest that NF-κB pathway plays an important role for DTP regrowth and drug resistance, and further that HOPX potentially converges with NF-κB pathway to regulate DTP progression.

### The function of HOPX on DTP progression is mediated by maintaining the compressed chromatin structure at early DTP stage

**Figure 6:**
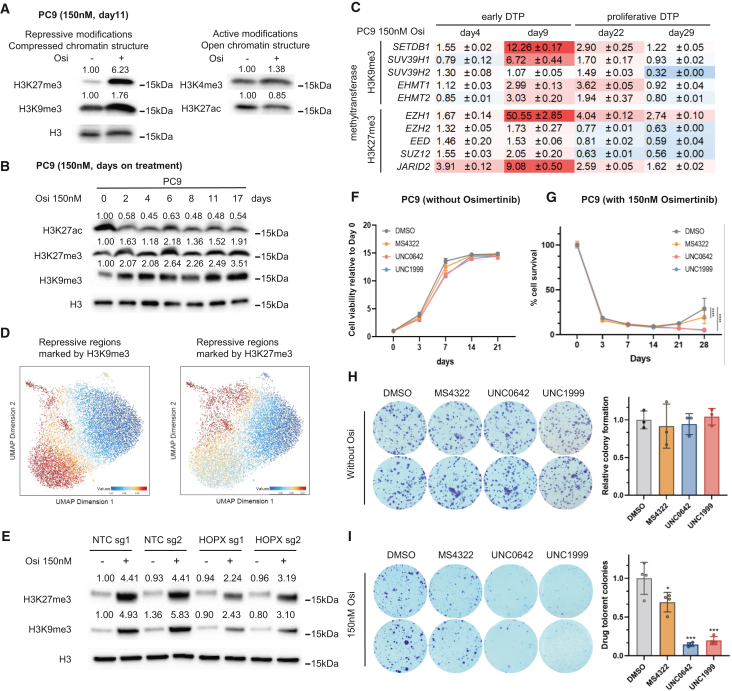
The function of HOPX on DTP progression is mediated by maintaining the compressed chromatin structure at early DTP stage (A) H3K27me3, H3K9me3, H3K27ac, and H3K4me3 levels measured by western blot on PC9 cells at 11 days post 150nM osimertinib treatment. (B) Western blot of H3K27me3, H3K9me3, and H3K27ac in PC9 cells at different days post 150nM osimertinib treatment. (C) mRNA levels of methyltransferases responsible for H3K9me3 (*SETDB1*, *SUV39H1*, *SUV39H2*, *EHMT1*, *EHMT2*) and H3K27me3 (*EZH1*, *EZH2*, *EED*, *SUZ12*, *JARID2*) at different time points (days 0, 4, 9, 22, 29) after PC9 cells were treated with 150nM osimertinib. qRT-PCR data were normalized to *GAPDH* level and the number indicate fold change relative to day0. Data are expressed as the mean ± standard error of three replicates for each condition. (D) Chromatin accessibility of repressive regions marked by H3K27me3 (*left*) or H3K9me3 (*right*) modifications projected to scATAC-seq UMAP. (E) Western blot of H3K27ac, H3K27me3 and H3K9me3 levels in PC9 cells transduced with non-target sgRNAs or HOPX sgRNAs with or without osimertinib treatment for 11 days. Quantification of the blot was measured by bands intensity and normalized to H3 and the untreated control group, and was labeled above the blot. (F) Cell growth of PC9 cells under 3μM PRMT5 inhibitor MS4322, G9a inhibitor UNC0642, or EZH2 inhibitor UNC1999 at indicated time points (normalized to day0). (G) Cell viability of PC9 cells under 150nM osimertinib and 3μM PRMT5 inhibitor MS4322, G9a inhibitor UNC0642, or EZH2 inhibitor UNC1999 at indicated time points (normalized to day0). (H) Colony formation assay on PC9 cells, at seeding density of 1,000 cells/well, with 3μM PRMT5 inhibitor MS4322, G9a inhibitor UNC0642, or EZH2 inhibitor UNC1999 starting on the second day of seeding. Two representative images were taken on 14 days after treatment per condition (*left*). Quantification of colony numbers (*right*). (I) Colony formation assay on PC9 cells, at seeding density of 150,000 cells/well under 150nm osimertinib with 3μM PRMT5 inhibitor MS4322, G9a inhibitor UNC0642, or EZH2 inhibitor UNC1999 starting on the second day of seeding. Two representative images were taken on 20 days after treatment per condition (*left*). Quantification of colony numbers (*right*). Data are presented as means ± S.D. For F) and G), comparisons between indicated groups were analyzed using two-way ANOVA followed by Tukey’s tests (*n* = 8). For H) and I), Statistical significance was determined using Kruskal-Wallis test. *n* = 3 in H) and *n* = 4 in I). ∗*p* < 0.05, ∗∗*p* < 0.01, ∗∗∗*p* < 0.001, ∗∗∗∗*p* < 0.0001.

DTP stage is dynamic and by definition reversible, which suggests a significant contribution from epigenomic change and regulation. It has been reported that DTP cells are marked by compressed chromatin structure.^21^ In our DTP system, we also found that DTP cells had markedly increased overall repressive markers H3K27me3 and H3K9me3, with a slight reduction in an active marker H3K27ac (Figure 6A), indicating the compressed chromatin structure is also critical for DTP cell development. We further investigated the temporal dynamics of compressed chromatin structure by testing repressive histone modifications (H3K27me3 and H3K9me3) at multiple time points during DTP development. The results showed that the histone modification change occurs at the very early DTP stage (at day 2) and maintains its level until the late DTP stage (at day 17) (Figure 6B). Similar H3K27me3 and H3K9me3 induction was also observed in sotorasib-induced NCI-H358 DTP model (Figure S5A). By evaluating the expression level of a series of histone modifying enzymes corresponding to H3K27me3 and H3K9me3, we found gene expression of various enzymes increased in early DTPs compared to non-treated cells, among which SETDB1 and EZH1 have the most significant induction, which decreased at the regrowth stage of DTP (Figure 6C).

To assess the heterogeneity of repressive chromatin state in the DTP subpopulations, we first obtained the repressive regions with H3K27me3 or H3K9me3 modifications through H3K27me3 ChIP-seq or H3K9me3 ChIP-seq, respectively on osimertinib-treated PC9 cells. We projected those regions to the scATAC-seq data from DTP cells and obtained the average accessibility to those repressive regions in each single cell, effectively inferring the level of repression (inaccessibility) imposed by H3K27me3 or H3K9me3 during DTP at a single cell level. We found that HOPX+ DTP sub-population has decreased accessibility on those regions, indicating this sub-population has more repressive histone modifications and more compressed chromatin structure (Figure 6D). The positive correlation of HOPX expression and repressive histone modifications in one specific DTP subpopulation prompted us to further investigate whether there is a direct regulatory role of HOPX on compressed chromatin dynamics in DTP cells. By comparing H3K27me3 and H3K9me3 levels in HOPX-deficient cells versus control cells under the treatment of osimertinib, we found that *HOPX* deletion reduced the induction of H3K27me3 and H3K9me3 by osimertinib treatment (Figure 6E). Similar regulatory role of HOPX on repressive chromatin structure was further confirmed in sotorasib-induced NCI-H358 DTP model (Figure S5B). Those results demonstrated that HOPX has a functional role for regulating compressed chromatin structure in DTP cells.

To identify targetable epigenomic regulators essential for maintaining the repressive DTP state, we performed a drug screening assay with a library containing 29 chemical compounds targeting various chromatin modifying enzymes. A gradient of various drug concentrations was applied together with the treatment with osimertinib. We identified seven candidate compounds to which PC9 cells were not sensitive when used alone, but exhibited response when combined with osimertinib (Figures S5C and S5D). We further verified the combinatorial treatment effect using cell viability assay and colony formation assay. The results showed that an EZH2 (which catalyzes H3K27me3) inhibitor UNC1999 or a G9A (which catalyzes H3K9me3) inhibitor UNC0642 or a PRMT5 (which catalyzes arginine methylation) inhibitor MS4322 alone did not influence cell growth rate or colony formation ability by itself (Figures 6F and 6H). Co-treatment with osimertinib and UNC1999 or UNC0642 significantly reduced DTP regrowth and colony formation. Additionally, co-treatment with osimertinib and MS4322 also suppressed DTP regrowth, although the effect was less pronounced compared to UNC1999 and UNC0642 (Figures 6G and 6I). These results demonstrate that the repressed chromatin structure is essential for DTP cells survival and targeting epigenomic regulators is a promising combinatorial treatment strategy for osimertinib resistance.

## Discussion

Molecularly targeted therapy is currently used as one of the key components of the standard treatment in lung cancer patients harboring specific oncogenic mutations such as *EGFR*, *KRAS*^*G12C*^, *ALK*, *RET*, and *ROS1*; however, tumor relapse is unfortunately an almost inevitable event for patients who receive these therapies, rendering these cancers essentially incurable. The reversible and transient DTP stage is potentially a major source of recurrence, by ensuring cancer cell survival from the stress, adapting to the rewired signaling activity, and re-entering cell cycle while developing features of acquired resistant. Therefore, identifying underlying mechanisms of DTP is a critical step forward to developing therapeutic strategies to suppress emergence of drug resistance effectively.

The research models currently being used to study DTP are not uniform, and there are generally two major approaches to induce drug resistance *in vitro*; one is starting with low concentration and gradually increasing drug dosage, which allows a larger cell population to survive, and the other is starting with a high dosage (e.g., at IC90). Although both can lead to acquired resistance in the end, the intermediate states and mechanisms might be molecularly different, thus, making the definition of DTP *in vitro* model somewhat controversial, and as such scientific findings based on the two models have been different. For example, Kashima et al. increase osimertinib concentrations from 10nM to 2000nM to induce drug resistance, and define cells treated at 30nM concentration as DTP.^33^ In this model, they demonstrated the heterogeneity of DTP states by single cell analysis where each cluster has specific marker genes including CD74 as a novel critical factor for DTP development.^33^ In another DTP model, Oren et al. used a high dosage (300nM) osimertinib to induce DTP stage and found that the DTP stage contains both fast cycling populations and slow cycling populations that have distinct transcriptional and metabolism programs.^25^ While both models represent important biology, in our study, to be closer to the concept of the residual disease in clinic when treatment is effective but it eventually leads to relapse, we continuously treated cells with drug at IC90 concentration, and define DTP stage when the growth curve has reached to and remain at nadir after drug treatment before they start to regrow.

The mechanism of DTP development is hypothesized to be either the consequence of selection of certain pre-existing populations resistant to stress or derived from stochastically selected surviving cells.^34^ In an EGFR inhibition study, it was reported that drug pre-treated persisters have higher frequency of persisters than drug naive cells, which is pre-determined by IRS1 phosphorylation level.^35^ Another study in colorectal cancer model demonstrated that DTP stage contains heterogeneous subtypes of stem-like cells, and homeostasis of the stem-like phenotypes is adaptive with capacity to undergo dynamic response to acute selective pressure and differentiate to a resistance state.^36^ We hypothesize that both mechanisms contribute to DTP development. Despite the various mechanisms explaining the origin of DTP, it has been well demonstrated that DTP stage is heterogeneous in multiple cancer types such as lung cancer,^37^^,^^38^ colorectal cancer,^36^ melanoma,^39^ etc. at transcriptome level by scRNA-seq. However, while those DTP subpopulations can be defined by clusters with specific molecular signatures, they were not usually separated distinctly, and the classification substantially varies across different models. We speculate that this is partially due to scRNA-seq reflecting transcriptomic signal which is relatively more fluid and dilutes the cancer cell intrinsic state, especially differentiation state, of DTP subpopulations. Thus, in this study, we focused on difference in the chromatin structure at epigenetic level to better capture cell lineage identity. Through scATAC-seq, we identified two major distinct sub-populations which likely represent different modes of persistence both in the EGFR inhibition model and the KRAS inhibition model. Meanwhile, we also characterized the heterogeneity of the same DTP stage at transcriptomic level by scRNA-seq and compared it with scATAC-seq classification. Both methods identified a similar number of unsupervised sub-clusters and showed two major sub-populations on UMAP. However, the separation of the two major sub-populations on UMAP is more obvious on scATAC-seq data than scRNA-seq data, suggesting classification based on chromatin structure is more clearly discernible than transcriptomic.

To better characterize the functional role of these two major sub-populations, we first filtered the irrelevant interference marker genes of each sub-population by projecting the marker gene to our previously established integrative LUAD network.^26^ This unique network was built based on causal gene regulatory network with Bayesian inference and allows us to extract and enrich functionally related marker genes and classified them based on their connection. Functionally, the pathway enrichment analysis indicated that one sub-population is governed by cell cycle signatures, while the other sub-population is enriched for organ development signatures. We posit that the evolution of drug resistance shares similarities with organ regeneration. Both require terminally differentiated cells regaining cell plasticity and serving as a progenitor cell to direct re-differentiation. In an autochthonous LUAD mice model, its precursor adenomas remain highly expressing alveolar marker genes.^15^ Coincidently, a recent study analyzing scRNA data from lung cancer biopsies from patients with residual disease (representing a DTP state) showed an elevated alveolar signature.^16^ The presence of alveolar signature in both tumor initiation and drug-induced resistance evolution suggests the connection between these two processes, i.e., the DTP stage can be viewed as a tumor re-initiation, where it allows future tumor development through modulating lung developmental pathways. One supporting evidence is from a study that showed YAP/TEAD activity was increased in DTP cells,^17^ while increasing evidence suggests Hippo/Yap plays important roles not only in lung progenitor cell differentiation^18^ but also in lung tumorigenesis.^19^ Thus, we speculate that lineage factors essential for lung development contribute to early DTP stage by directing the cells into the specific lineage state pertinent to DTP characteristics. Therefore, we focused on alveolar signatures for the two major DTP subpopulations, and found a lung lineage factor HOPX is commonly enriched in one of the sub-populations also enriched with developmental pathways across two independent LUAD DTP models.

Our scATAC-seq and bulk gene expression analyses revealed some discrepancies in HOPX expression dynamics. While scATAC-seq performed on Day 11 showed HOPX enrichment in a specific DTP subpopulation, bulk gene expression data indicated that HOPX levels peaked at Day 2 and returned to baseline by Day 11. This difference may arise from chromatin accessibility reflecting a more stable lineage state, whereas gene expression is more dynamic, influenced by transcription, translation, and protein stability. Additionally, bulk RNA measurements represent an average across all cells, while scATAC-seq captures a specific sub-population, making temporal variations in subpopulation proportions another possible contributing factor. Another possible indication of dynamic changes in the proportions of the two DTP subpopulations during the DTP process is shown in Figure S4C. We observed that bulk YAP levels initially decreased and then increased, while HOPX levels exhibited an inverse trend, first increasing and then decreasing. As mentioned in Figure 5A, YAP/TEADs is more enriched in the HOPX-negative sub-population. This complementary pattern may reflect protein-level changes or shifts in the relative proportions of the two subpopulations over time. Together, these observations suggest the possibility of a dynamic interplay between these subpopulations contributes to the complexity of DTP development and highlights the need for subpopulation-specific studies in future research. Nevertheless, we demonstrated that the level of HOPX can be a biomarker for early DTP stage and is functionally essential for DTP development. Coincidently, a very recent study also found that tumor cells entered to a quiescent alveolar type 1 cell like stage under KRAS inhibition, which give rise to drug resistance differentiation potential and facilitate resistance process in both genetically engineered mouse models and patient-derived xenografts.^40^ Conceptually, these two studies simultaneously uncovered a novel idea that views drug resistance evolution as a tumor re-initiation that is controlled by their native regenerative potential. Targeting lung lineage developmental genes such as HOPX to prevent drug resistance may be a previously unrecognized cancer vulnerability during effective treatment.

To target DTP and prevent tumor relapse, many signal pathways have already been identified to participate in drug resistance progress, including transcriptional regulation such as IGF-1R,^41^ WNT/β-catenin,^42^ FGFR3,^43^ YAP/TEADs^17^; epigenomic modification such as KDM5A^44^; and metabolic remodeling such as ALDH,^45^ GPX4^46^ etc. In this study, instead of focusing on the expression level of these programs, we first investigated chromatin accessibility in the two DTP sub-populations from scATAC-seq, which reflect additional critical molecular features including the transcriptional factor activity. We found that HOPX+ sub-population is enriched for NF-κB activity, while HOPX-sub-population is enriched for YAP/TEADs activity. Both pathways have been reported to promote acquired drug resistance, especially YAP/TEAD pathways have been widely reported to regulate tumor cell regrowth after drug treatment. This feature coincides with our findings that YAP/TEADs enriched in one of the sub-populations that is HOPX- and has proliferative signatures. For the other HOPX+ sub-population where NF-κB is enriched, we found HOPX level influence NF-κB activity under DTP condition, indicating HOPX regulation of DTP development is directly or indirectly converging with NF-κB pathway activity. Our data also suggest that the induction of NF-κB activation occurred at early DTP stage and is a relatively early event during acquired resistance process, and that combinational therapy of NF-κB inhibitor and osimertinib could be an option to suppress DTP development.

Given the reversible nature of DTP stage, epigenomic regulation is another attractive mechanism to target DTP and prevent resistance. A main feature of the DTP stage is a global repressive chromatin state characterized by increased repressive histone H3 modifications, which is especially prominent at LINE-1 elements.^21^ Treatment with HDAC inhibitors, which leads to a less compacted chromatin state, also causes a reduced DTP growth, indicating the importance of heterochromatin in maintaining DTP survival.^21^ In this study, we demonstrated an increased repressive-chromatin modifications both in osimertinib- and sotorasib-induced DTP model. The chromatin modification of DTP is well studied at bulk level in different DTP models, but little is described in terms of their heterogeneity. Ideally, epigenetic modification heterogeneity could be assessed with single cell technologies such as a recently developed technique scCUT&Tag, but it has not been widely utilized. Here, we devise a creative analytical approach using H3K27me3 and H3K9me3 ChIP-seq data and scATAC-seq data, by projecting accessibility on potentially repressive sites effectively reveal repressive profiles per cell, which reflect those modification at single cell resolution. Our result demonstrated that the repressive chromatin structure is heterogeneous in DTP stage. The HOPX+ sub-population is enriched with repressive histone modification since it is less accessible at the repressive modification regions determined by H3K27me3 and H3K9me3 ChIP-seq, indicating a more compressed chromatin structure and less transcription activity, which is consistent with the known concept of DTP heterochromatin. In contrast, the HOPX-negative subpopulation which is dominated by cell cycle and proliferation pathways has relatively more open chromatin structure, indicating they are preparing to increase transcriptional activity for later regrowth. HOPX deletion also inhibited the overall level of H3K27me3 and H3K9me3 in DTP cells, suggesting HOPX either directly regulate chromatin modification or indirectly influence the chromatin structure through determining the lineage state of DTP.

Taken together, we characterized the heterogeneity and plasticity of DTP stage at epigenetic level and identified two distinct sub-populations with different functional enrichment. We found that lung lineage factor HOPX is commonly enriched in one of the sub-populations across two independent DTP models. Further functional studies demonstrated that HOPX is essential for the development of DTP and we were able to modulate DTP progression through suppressing NF-κB pathway and repressive chromatin structure.

### Limitations of the study

In this study, the two abundant sub-clusters were identified and characterized in scATAC-seq and scRNA-seq as they exhibited more distinct subpopulation identities and provided clearer pathway enrichment results. Yet, the lack of effective methods to separate them limits our ability to study their functional differences. Consequently, our investigation of HOPX expression and function has been conducted at the bulk level, preventing direct validation of gene expression changes within specific subpopulations. Similarly, genes such as CLDN18, and NKX2-1 were identified as differentially enriched in specific clusters, but bulk expression analysis only confirms their overall upregulation in DTP cells after treatment, without specifying which subpopulation is responsible. Future studies could focus on identifying subpopulation-specific surface markers, which would enable the isolation of these clusters via flow cytometry. This approach would allow for a more precise investigation of the functional roles and interactions of distinct DTP subpopulations, providing deeper insights into their contributions to DTP progression and therapeutic resistance. In addition, multiple smaller sub-clusters were also identified in this study, while preliminary functional analysis did not reveal pathways strongly associated with tumor resistance, leaving their potential roles uncertain. Current experimental limitations prevent the isolation and characterization of individual clusters, restricting our ability to validate their functional contributions. Further research is needed to determine whether these smaller clusters represent functionally relevant subpopulations with previously unrecognized roles in resistance, which remains an important direction for future investigation.

While HOPX is identified as a potential regulator of DTP development, its physiological significance in development and constitutive expression as a key marker of alveolar type 1 cells raise concerns about potential toxicity if directly targeted. As a result, HOPX itself may not be a viable therapeutic target, and further investigation is needed to elucidate its regulatory mechanisms and identify clinically actionable pathways that can be validated *in vivo*. Currently, the role of HOPX in drug resistance evolution remains largely unexplored. In this study, we provide preliminary insights into its potential mechanisms, including its association with the NF-κB pathway and chromatin organization. However, the precise regulatory mechanisms remain unclear. One possibility is that HOPX influences the overall DTP state, which in turn affects pathways such as NF-κB activation. Alternatively, HOPX may regulate NF-κB signaling indirectly through intermediate pathways, though it is unlikely to be the sole regulatory factor. Considering that HOPX is a transcription factor, it likely functions by binding directly to DNA or interacting with specific proteins. Future studies should focus on identifying its direct regulatory targets, such as through HOPX ChIP-seq or alternative approaches to map its DNA binding sites and target genes. Additionally, proteomic studies could help uncover protein-protein interactions that mediate HOPX function in DTP cells. These insights will be essential for understanding HOPX-mediated regulation and exploring potential therapeutic strategies targeting downstream effectors.

## Resource availability

### Lead contact

Further information and requests for resources and reagents should be directed to the lead contact, Hideo Watanabe (Hideo.Watanabe@mssm.edu).

### Materials availability

This study did not generate new unique reagents.

### Data and code availability

• ChIP-seq, scRNA-seq and scATAC-seq data generated in this study were deposited at the Gene Expression Omnibus (https://www.ncbi.nlm.nih.gov/geo/) under accession number GSE275183, GSE275199 and GSE275305 respectively.
• This paper does not report original code.
• Any additional information required to reanalyze the data reported in this paper is available from the lead contact upon request.

## Acknowledgments

Y.T. is supported by EGFR Resisters and Lung Cancer Research Foundation research award (2021-Tian-1688). H.W. has been supported by the American Lung Association of the Northeast Lung Cancer Discovery Award (LCD-504985, LCD-92032011), 10.13039/100000005Department of Defense (W81XWH-19-1-0613), and 10.13039/100000002NIH (R01CA240342). We appreciate Jian Jin for providing the chemical drug library for the screening assay. This work utilized the NMR Spectrometer Systems at Mount Sinai acquired with funding from the NIH’s SIG grants 1S10OD025132 and 1S10OD028504. We thank Yifei Sun, Shuhui Liu, Ranran Kong, Takashi Sato, Ayushi S. Patel, Abhilasha Sinha, Charles A. Powell, and Susumu Kobayashi for helpful discussions and technical assistance. We acknowledge the Genomics Core Facility at ISMMS for the world-class next generation sequencing platform. This work was supported in part through the computational and data resources and staff expertise provided by Scientific Computing and Data at the Icahn School of Medicine at Mount Sinai and supported by the Clinical and Translational Science Awards (10.13039/100016220CTSA) grant UL1TR004419 from the 10.13039/100006108National Center for Advancing Translational Sciences. Research reported in this publication was also supported by the Office of Research Infrastructure of the National Institutes of Health under award number S10OD026880 and S10OD030463. The content is solely the responsibility of the authors and does not necessarily represent the official views of the National Institutes of Health.

## Author contributions

Y.T. and H.W. designed the experiments. Y.T., R.B., and F.J., performed the experiments. E.P. and G.L. performed the drug screening assay. J.J., Y.S., K.P., and H.U.K. developed and provided the chemical drug library. Y.T., R.B., S.Y., J.Z., B.H., and H.W. analyzed the data. Y.T. and H.W. wrote the paper. All authors edited and critically reviewed the paper and agree to the final version of the manuscript.

## Declaration of interests

J.J. is a cofounder and equity shareholder in Cullgen, Inc., a scientific cofounder and scientific advisory board member of Onsero Therapeutics, Inc., and a consultant for Cullgen, Inc., EpiCypher, Inc., Accent Therapeutics, Inc, and Tavotek Biotherapeutics, Inc. The Jin laboratory received research funds from Celgene Corporation, Levo Therapeutics, Inc., Cullgen, Inc. and Cullinan Oncology, Inc.

All other authors declare no competing interests.

## STAR★Methods

### Key resources table

**Table:** 

REAGENT or RESOURCE	SOURCE	IDENTIFIER
**Antibodies**		

Anti-HOPX antibody produced in rabbit	Sigma-Aldrich	Cat#HPA030180; RRID: AB_10603770
Anti-Actin antibody produced in rabbit	Sigma-Aldrich	Cat#A2066; RRID: AB_476693
Anti-Vinculin Antibody	Sigma-Aldrich	Cat#V9131; RRID: AB_476693
YAP Antibody	Cell Signaling Technology	Cat#4912; RRID: AB_2218911
NF-κB p65 (D14E12) XP® Rabbit mAb	Cell Signaling Technology	Cat#8242; RRID: AB_10859369
Phospho-NF-κB p65 (Ser536) (93H1) Rabbit mAb	Cell Signaling Technology	Cat#3033; RRID: AB_331284
Tri-Methyl-Histone H3 (Lys27) (C36B11) Rabbit mAb	Cell Signaling Technology	Cat#9733; RRID: AB_2616029
Histone H3 (D1H2) XP® Rabbit mAb	Cell Signaling Technology	Cat#4499; RRID: AB_10544537
Anti-Histone H3 (tri methyl K9) antibody	Abcam	Cat#ab8898; RRID: AB_306848
Anti-Histone H3 (acetyl K27) antibody	Abcam	Cat#ab4729; RRID: AB_2118291
Anti-mouse IgG, HRP-linked Antibody	Cell Signaling Technology	Cat#7076; RRID: AB_330924
Anti-rabbit IgG, HRP-linked Antibody	Cell Signaling Technology	Cat#7074; RRID: AB_2099233
Goat anti-Rabbit IgG (H + L) Secondary Antibody, FITC	Thermo Fisher Scientific	Cat#65-6111; RRID: AB_2533966

**Bacterial and virus strains**		

One Shot™ Stbl3™ Chemically Competent E. coli	Invitrogen	Cat#C737303

**Chemicals, peptides, and recombinant proteins**		

Osimertinib (AZD9291)	Selleck Chemicals	Cat#S7297
Sotorasib (AMG510)	ChemGood	Cat#C-1499
BAY11-7082	Selleck Chemicals	Cat#S2913
MS4322	Jian Jin laboratory	N/A
UNC0642	Jian Jin laboratory	N/A
UNC1999	Jian Jin laboratory	N/A

**Critical commercial assays**		

RNeasy kit	QIAGEN	Cat#79656
QuantiTect Reverse Transcription Kit	QIAGEN	Cat#205313
EvaGreen dye	Biotium	Cat#31000
ROX reference dye	Invitrogen	Cat#12223012
KAPA HiFi HotStart ReadyMix PCR Kit	KAPA Biosystems	Cat# KR0370
AlamarBlue™ Cell Viability Reagent	Thermo Fisher	Cat#DAL1025
Western Blotting Luminol Reagent	Santa Cruz Biotechnology	Cat#sc-2048
TransIT®-Lenti Transfection Reagent	Mirus Bio	Cat#MIR6604
Dynabeads_Protein_G	Life Technologies	Cat#10003D
Agilent High Sensitivity DNA Kit	Agilent Technologies	Cat#5067-4626 RUO
NEBNext Ultra II DNA Library Prep Kit	New England BioLabs	Cat#E7645
10x Genomics Reagent kits	10x Genomics	https://www.10xgenomics.com/

**Deposited data**		

Bayesian network of LUAD	Yoo et al.^26^	Yoo et al.^26^
ChIP-seq	This study	GEO: GSE275183
sc-RNAseq	This study	GEO: GSE275199
sc-ATACseq	This study	GEO: GSE275305

**Experimental models: Cell lines**		

HEK293T	Laboratory stocks	RRID: CVCL_0063
PC9	Laboratory stocks	RRID: CVCL_B260
NCI-H358	Laboratory stocks	RRID: CVCL_1559

**Oligonucleotides**		

Primers for RT-qPCR	Origene	See Table S1
sgRNAs targeting HOPX	Brunello library	See Table S2
non-target sgRNAs	Gecko library v2	See Table S2

**Recombinant DNA**		

Plasmid: lentiCas9-Blast	Addgene	Cat#52962
Plasmid: lentiGuide-Puro	Addgene	Cat#52963

**Software and algorithms**		

Prism9	GraphPad	https://www.graphpad.com/
ImageJ	ImageJ	https://imagej.nih.gov/ij/
Image Lab Software	Bio-Rad	https://www.bio-rad.com/
Axio Imager Z2	Zeiss	https://www.zeiss.com/microscopy/en/products/light-microscopes
Metascape	Zhou et al.^47^	https://metascape.org

### Experimental model and subject details

#### Cell lines and culture

Experimental cell lines including PC9 (RRID: CVCL_B260, gender: male), NCI-H358 (RRID: CVCL_1559, gender: male), HEK293T (RRID: CVCL_0063, gender: female) were consistently maintained in our laboratory. PC9, NCI-H358 were maintained in RPMI1640 (Gibco) with 10% Fetal Bovine Serum (FBS) and 1% penicillin–streptomycin (Gibco). HEK293T cells were maintained in DMEM (Gibco) with 10% FBS and 1% penicillin–streptomycin (Gibco). All cells were cultured at 37°C in incubator with 5% CO_2_. All cell lines have been authenticated by routine mycoplasma contamination testing.

#### DTP cell derivation

DTP cells were derived using an IC_90_ drug concentration, specifically for PC9 cell with the treatment of 150nM Osimertinib and for NCI-H358 with the treatment of 500nM Sotorasib. Culture medium containing drug was replaced every 3–4 days. Bright field images were captured at indicated time point by microscope using 10× objective lens.

### Method details

#### Sample preparation for single cell sequencing

PC9 DTP cells were collected 11 days after the start of drug treatment and NCI-H358 DTP cells were collected 8 days after the start of drug treatment. PC9-untreated and NCI-H358-untreated cells were collected in parallel as controls under the same culture conditions without drug exposure. For each sample, a million cells were resuspended in Phosphate-buffered saline (PBS) with 0.04% Bovine Serum Albumin (BSA). For scATAC-seq, nuclei isolation was performed following the 10x Genomics Nuclei Isolation for Single Cell ATAC Sequencing protocol, and libraries were generated using the Chromium Next GEM Single Cell ATAC Reagent Kits (10x Genomics). For scRNA-seq, samples were processed using the Chromium Single Cell 3′Reagent Kits (10x Genomics) according to the manufacturer’s instructions. Both scATAC-seq and scRNA-seq were performed at the Icahn School of Medicine at Mount Sinai Genomics Core Facility, and libraries were sequenced on NovaSeq 6000 (Illumina).

#### Single cell sequencing data analysis

Sequencing fragments were first aligned to a reference hg38 genome, and removed duplicate fragments by CellRanger. ArchR^48^ was used to analyze the data. Specifically, open chromatin regions were detected and calculated for the enrichment of reads in open chromatin regions, or features, per cell. Cells were filtered out by potential doublets (enrichment ≥1), the number of fragments (≥100), and transcription start site (TSS) enrichment (≥4). The features versus cell matrix was then be used to select variable features, dimensionality reduction, and graph-based clustering. Gene accessibility score was calculated after Iterative Latent Semantic Indexing (LSI) transformation and Markov Affinity-based Graph Imputation of Cells (MAGIC) imputed smoothened scores were projected on UMAP. Motif enrichment in open chromatin regions was evaluated from the peaksets called by MACS2 and annotated by CIS-BP database.^49^ Motif enrichment was similarly projected to UMAP. Marker features for a cluster were selected at FDR ≤ 0.1 and an absolute Log2FC ≥ 0.5 by comparing major clusters as indicated in the main text.

For H3K27me3 and H3K9me3 accessibility analyses, first the enriched broad domains for each histone modification was called by MACS2. Aggregate signals in the enriched domains that are normalized with +/− 2kb flanking regions of each domain were calculated per each cell. Then, MAGIC imputed smoothened scores were projected on UMAP.

#### Chromatin Immunoprecipitation sequencing assay

Chromatin Immunoprecipitation sequencing (ChIP-seq) was performed as described previously.^50^ PC9 cells treated with 150nM Osimertinib were fixed with 1% formaldehyde for 8 min. After quenching and cell lysis, chromatin was sonicated for 6 min using QSONICA. Protein G magnetic beads (Life Technologies) and antibodies against H3K9me3 (Abcam, Cat#ab8898, 1:500 dilution) or H3K27me3 (CST,Cat#9733, 1:500 dilution) were used to immunoprecipitated the DNA fragment. Up to 10 ng DNA was used for the library construction using NEBNext Ultra II DNA Library Prep Kit (New England BioLabs). Sequencing was performed on NextSeq 500 (Illumina).

#### Bayesian network and gene ontology analysis

The Integrative network of LUAD was built by our group as previously described.^26^ To identify subnetworks enriched for DTP sub-clusters, the signature gene was overlaid on the network, and neighboring nodes within two layers for each signature were selected to obtain a list of functional enriched signature genes. Pathway enrichment analysis of the functional enriched signature genes were analyzed with Metascape^47^ under GO Biological Processes terms.

#### Quantitative reverse transcription PCR

Total RNA was extracted using RNeasy kit (Qiagen), and converted to cDNAs using QuantiTect Reverse Transcription Kit (Qiagen) according to the manufacturer’s instructions. qRT-PCR was performed using the mix of EvaGreen dye (Biotium), ROX reference dye (Invitrogen), and Kapa Taq DNA Polymerase (KAPA Biosystems). The results were normalized to a housekeeping gene *GAPDH* and the control group. All primers were synthesized by Integrated DNA Technologies, and primer sequences were listed in Table S1.

#### Western blot

Samples from whole-cell lysate (150 mM NaCl, 50 mM Tris-HCl at pH 8.0, 1% NP-40, 0.5% Na deoxycholate, 0.1% SDS, 1xprotease inhibitors) were subjected to electrophoresis on an SDS-PAGE (Bio-Rad). Proteins were transferred onto a polyvinylidene difluoride membrane (Thermo Fisher Scientific) and blocked with 5% milk for 1 h. Membranes were incubated with primary antibodies overnight at 4°C, followed by incubation with HRP-conjugated secondary antibodies for 1 h at room temperature. The antibody-labeled proteins were detected using Western Blotting Luminol Reagent (Santa Cruz Biotechnology) and imaged with Image Lab Software (Bio-Rad). Primary antibodies against HOPX (Cat#HPA030180, 1:1000 dilution), Vinculin (Cat#V9131, 1:1000 dilution), Actin (Cat#A2066, 1:1000 dilution) were from Sigma-Aldrich. Antibodies against YAP (Cat#4912, 1:1000 dilution), p65 (Cat#8242, 1:1000 dilution), p-p65 (Cat#3033, 1:1000 dilution), H3K27me3 (Cat#9733, 1:1000 dilution), and Anti-H3 (Cat#4499, 1:1000 dilution) were from Cell Signaling Technologies. Anti-H3K9me3 (Cat#ab8898, 1:1000 dilution) and Anti-H3K27ac (Cat#ab4729, 1:1000 dilution) antibodies were from Abcam, Secondary antibody HRP-linked anti-mouse IgG and anti-rabbit IgG were from Cell Signaling Technologies at 1:5000 dilution.

#### Immunofluorescence

PC9 cells were seeded on glass coverslips and cultured for 24 h then treated with 150nM Osimertinib for indicated time. Cells were fixed with 4% paraformaldehyde for 30 min, permeabilized with 0.2% Triton X-100 for 10 min, blocked with 5% goat serum for 1 h, incubated with primary antibodies (1:100 dilution) overnight at 4°C, followed by incubation with FITC-conjugated secondary antibodies (1:200 dilution) for 1 h at room temperature. Nuclei were stained with 4,6-diamidino-2-phenylindole (DAPI) for 2 min at room temperature. Images were captured using Axio Imager Z2 (Zeiss). The cytoplasmic versus nucleus localized HOPX were quantified by ImageJ software. HOPX N/C ratio was calculated by the mean nuclear intensity divided by the mean cytoplasmic intensity of FITC channel.

#### CRISPR-Cas9 gene deletion and lentivirus infection

Cells stably expressing human codon-optimized S. pyogenes Cas9 expression were generated by infection with the lentiCas9-Blast plasmid (a gift from Feng Zhang, Addgene #52962). sgRNAs targeting HOPX (selected from Brunello library) and non-target sgRNAs (selected from the Gecko library v2) were synthesized by Integrated DNA Technologies and listed in Table S2 sgRNAs were cloned into lentiGuide-Puro (Addgene #52963) at Esp3I site. For lentivirus production, HEK293T cells were transfected with 10 μg of lentiviral plasmids, 7.5 μg of psPAX2, and 2.5 μg of pMD2.G, utilizing TransIT-Lenti (Mirus) in accordance with the manufacturer’s guidelines. After 48 h, viral supernatants were collected, passed through a 0.45 μm filter, and stored at −80°C for further use. Cells were first infected with the lentiCas9-Blast lentivirus and then selected with blasticidin (8 μg/mL for 10 days). Cas9 expressing cells were then infected with lentiGuide-Puro lentivirus containing HOPX sgRNA and selected with Puromycin (2 μg/mL for 5 days). Knock out efficiency was tested by Western blot at bulk level.

#### Cell viability assay and drug screening assay

For cell growth assay without Osimertinib or Sotorasib, 1500 cells were seeded into 96-well plates with 12 replicates per condition. For cell survival assay with Osimertinib or Sotorasib, 25000 cells were seeded into 96-well plates with 12 replicates per condition. Indicated concentration of drugs were added after overnight attachment. Fresh media with drug were replaced every 3–4 days. Cells plates were washed twice with PBS before testing the cell viability at indicated time points. Cell viability was assayed with alamarBlue Cell Viability Reagent (Thermo Fisher) and fluorescence at 585 nm was measured on a Spectra Max3 plate reader (Molecular Device) according to the manufacturer’s protocol at excitation of 555 nm.

For drug screening assay, 2,000 cells were seeded into 384 well plate (3 plates per condition) and treated with indicated drugs at 6 different concentrations (with the highest at 10uM serially diluted by the factor of 3) in combination with different Osimertinib concentration (0nM, 2nM, 4nM, and 3μM). Fresh media with drug were replaced at day5, and images were captured at day9. Confluency was calculated using Operetta CLS high-content analysis system.

#### Colony formation assay

Cells were seeded in six-well plates at a concentration of 150,000 per well and two wells per condition. Indicated drugs were added on the next day after seeding, and fresh medium with drug was replaced every 3–4 days. After 20 days of incubation, colonies were fixed with 4% paraformaldehyde and stained with crystal violet. Images were captured and adjusted uniformly to 7-inch diameter for each well. Colony numbers were quantified using 0.015-infinity (inch^2^) as size threshold by ImageJ software. The initial seeding density (150,000 cells per well) was selected to prevent excessive colony merging, allowing for clear quantification at the final time point after colony regrowth. This differs from the DTP sampling method in single-cell sequencing experiments, which required a higher initial density to ensure sufficient cell numbers for analysis at the lowest survival rate during the DTP phase. Despite this difference in seeding strategy, both assays followed the same drug dosing schedule and concentration to maintain consistency in DTP induction.

### Quantification and statistical analysis

Bar graphs and line graphs are presented as mean ± s.d. For comparisons with small sample sizes (n=<6), the Mann-Whitney test was applied to compare the statistical significance between two groups. Kruskal-Wallis was employed to analyze difference across multiple groups. For large sample size (*n* > 6), a two-tailed, unpaired Student’s t test was applied. *p* < 0.05 was considered to be significantly different. GraphPad Prism software was used to plot and analyze data. The correlation between HOPX+ sub-population across different models was analyzed by hypergeometric test.

## Supplemental information

Document S1. Figures S1–S5, Tables S1, S2, and S4

Table S3. Signature genes of PC9 DTP clusters revealed by scATAC-seq, related to Figure 1

Table S5. Signature genes of NCI-H358 DTP clusters revealed by scATAC-seq, related to Figure 2

Table S6. Bayesian regulatory network node genes derived from signature genes of NCI-H358 DTP clusters, related to Figure 2
