# Supplementary material for: Epigenomic analysis identifies DTP subpopulation using HOPX to develop targeted therapy resistance in lung adenocarcinoma
Source: iScience. 2025 Apr 13;28(5):112387. doi: 10.1016/j.isci.2025.112387 (PMC12063144; doi:10.1016/j.isci.2025.112387)
Supplement: Document S1. Figures S1–S5, Tables S1, S2, and S4 [file mmc1.pdf]

iScience, Volume *28*

Supplemental information

Epigenomic analysis identifies DTP subpopulation using HOPX to develop targeted therapy resistance in lung adenocarcinoma

Yang Tian, Reshmee Bhattacharya, Seungyeul Yoo, Feng Jiang, Eric Park, Genesis Lara Granados, Yudao Shen, Kwang-Su Park, Husnu Umit Kaniskan, Jian Jin, Benjamin D. Hopkins, Jun Zhu, and Hideo Watanabe

Figure S1

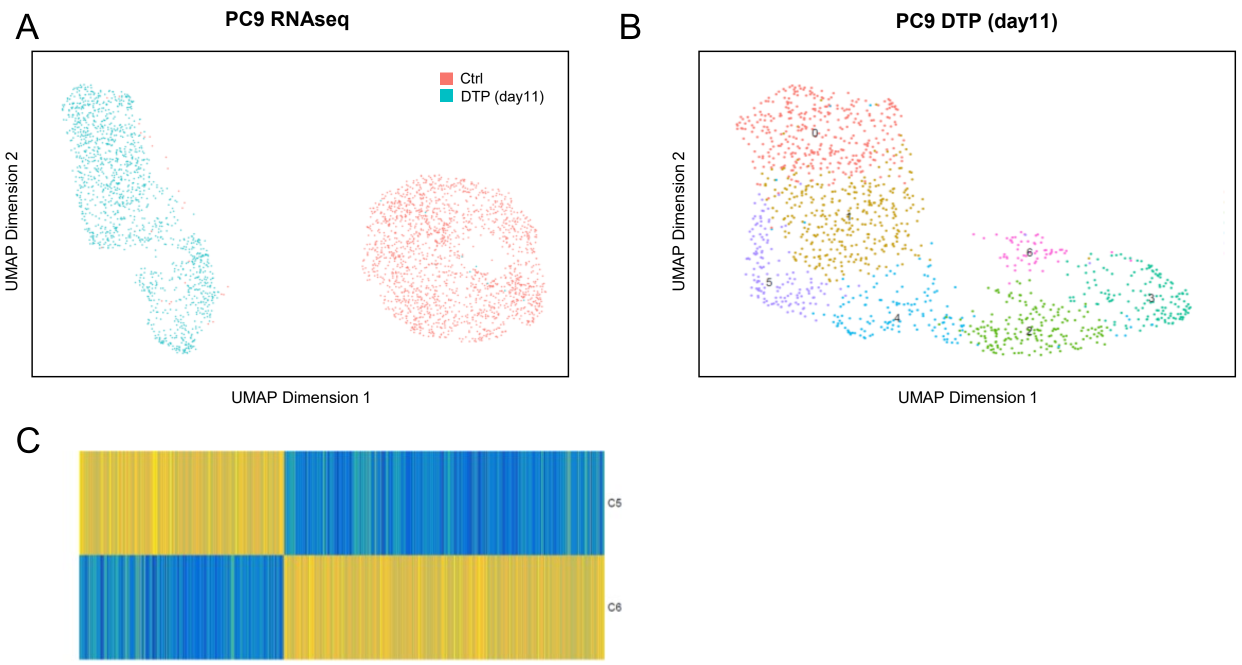
A
PC9 RNAseq
Ctrl
DTP (day11)
UMAP Dimension 2
UMAP Dimension 1
B
PC9 DTP (day11)
UMAP Dimension 2
UMAP Dimension 1
C
C5
C6

Figure S1. The heterogeneity of Osimertinib-induced DTP stage revealed by scATAC-seq.

A-B). Heterogeneity of DTP cells (150nM, day 11) revealed by UMAP representation of scRNA-seq data. A): Cells colored by sample; control (orange) and Osimertinib-induced DTP (green). B): Cells colored by unsupervised clusters of Osimertinib-treated samples. **C).** Heatmap showing gene accessibility of 430 cluster-5 signature genes and 619 cluster-6 signature genes in PC9-DTP cells revealed by marker gene analysis of the scATAC data.

Figure S2

A

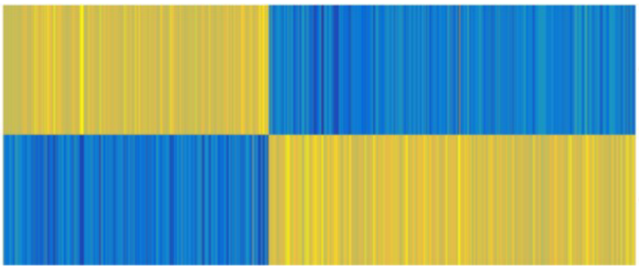

B

H358 DTP-cluster3

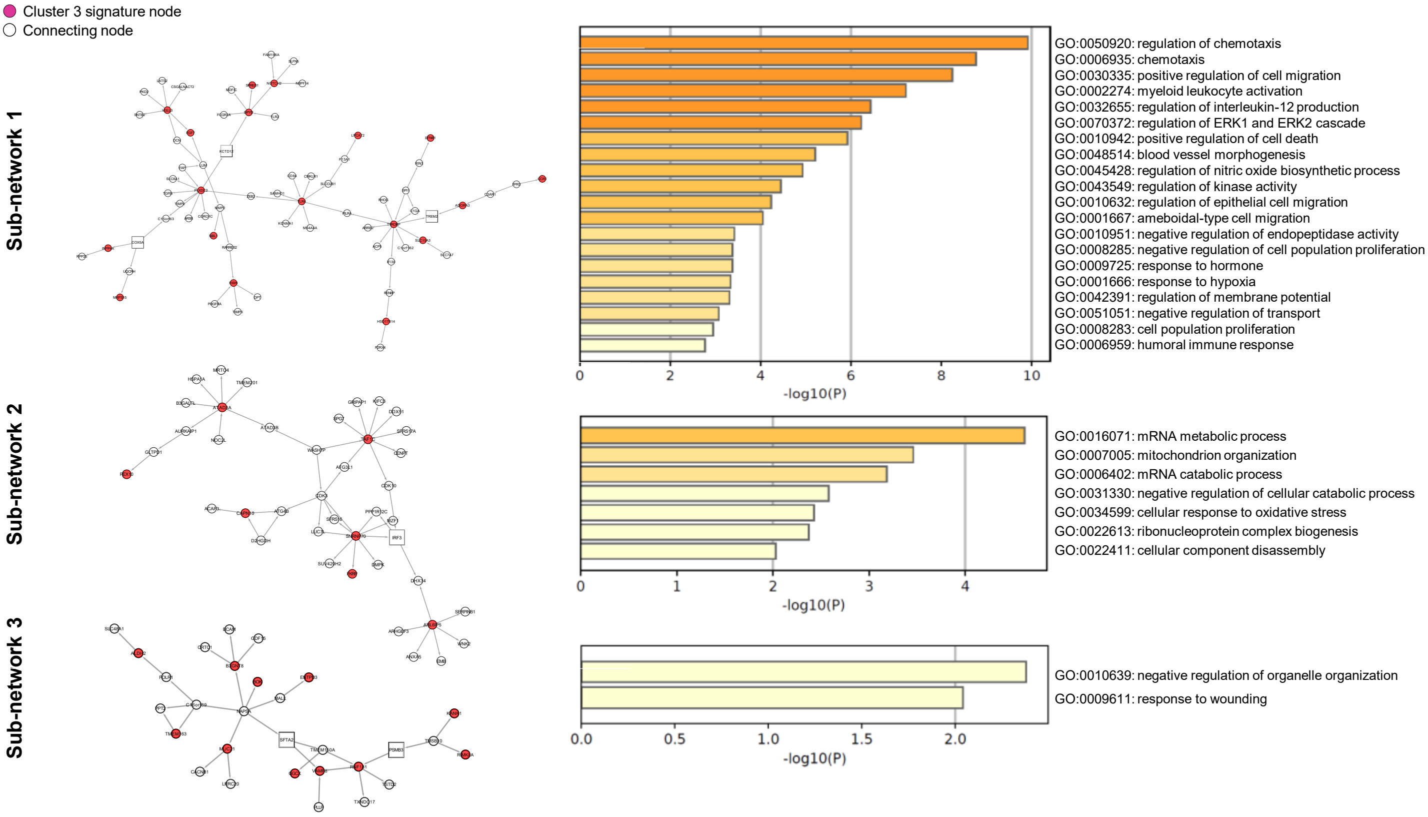

Figure S2

C

H358 DTP-cluster4

Cluster 4 signature node
Connecting node

Sub-network 1

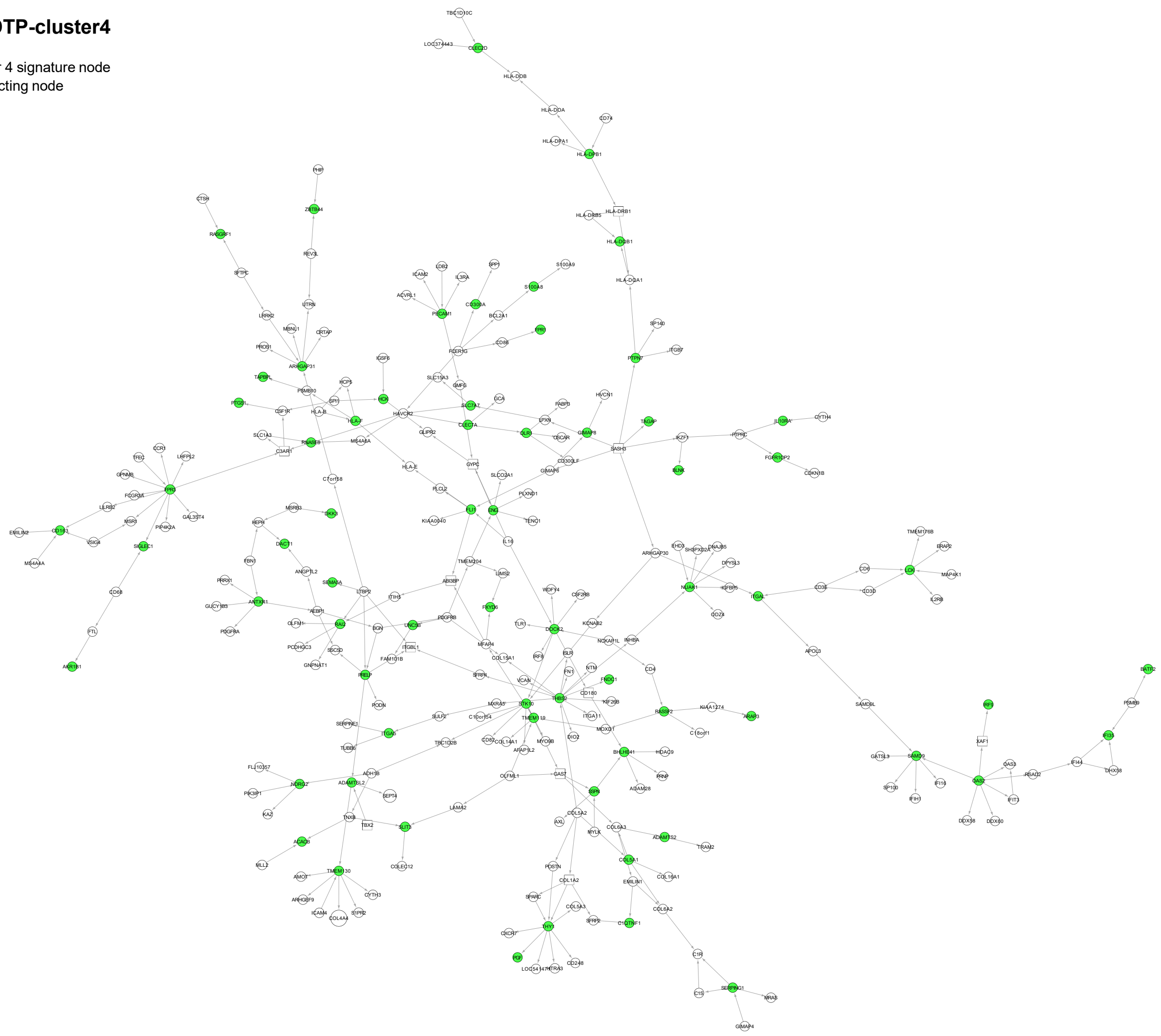

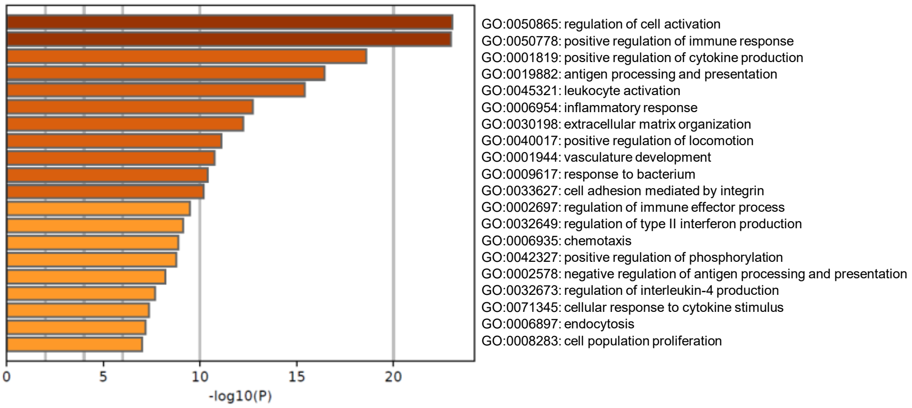

Sub-network 2

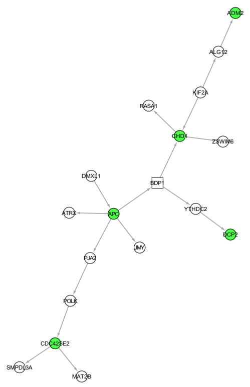

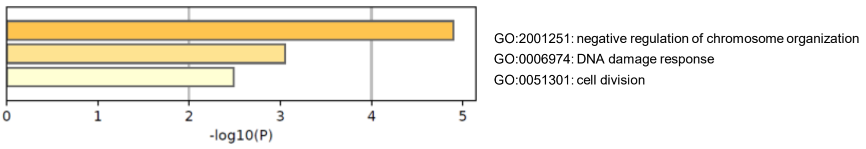

Figure S2. The heterogeneity of Sotorasib-induced DTP stage revealed by scATAC-seq.

A). Heatmap showing gene accessibility of 1,171 cluster-3 signature genes and 1,385 cluster-4 signature genes in NCI-H358-DTP cells revealed by marker gene analysis of the scATAC data. **B)**. Subnetwork with gene node connections at one layer when cluster 3 signature (Table S5) is projected to LUAD Bayesian network. Pink nodes represent cluster 3 signature genes, and nodes indicated in square are key drivers of the subnetwork (see Methods). All nodes (genes) of subnetworks were listed in Table S6. Gene ontology (GO) analysis of sub-networks nodes (genes) of cluster 3 (Table S6). **C)**. Subnetwork with node connections at one layer when cluster 4 signature (Table S5) is projected to LUAD Bayesian network. Green nodes represent cluster 4 signature genes, and nodes indicated in square are key drivers of the subnetwork (see Methods). All nodes (genes) of subnetworks were listed in Table S6. Gene ontology (GO) analysis of sub-networks nodes (genes) of cluster 3 (Table S6).

Figure S3

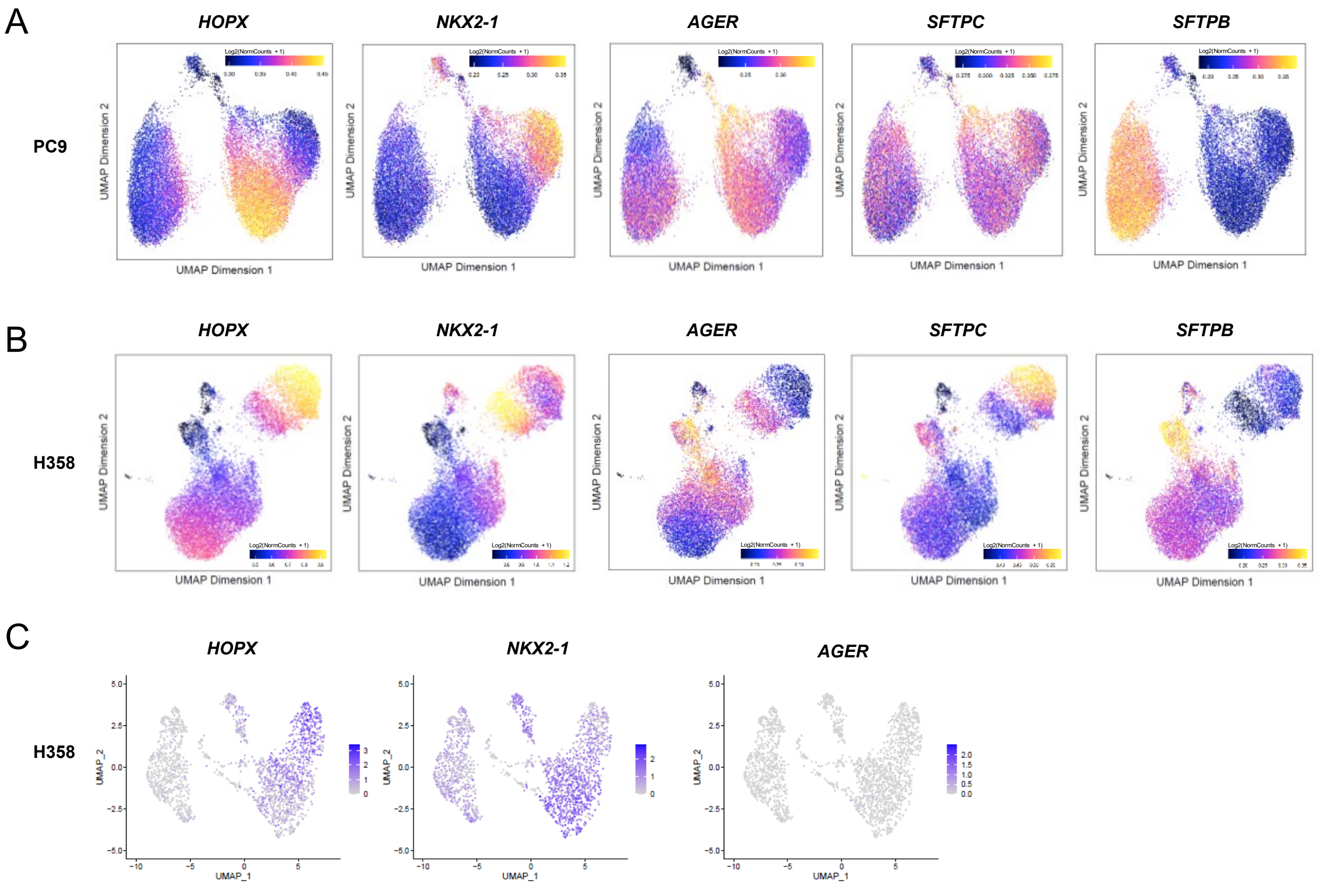

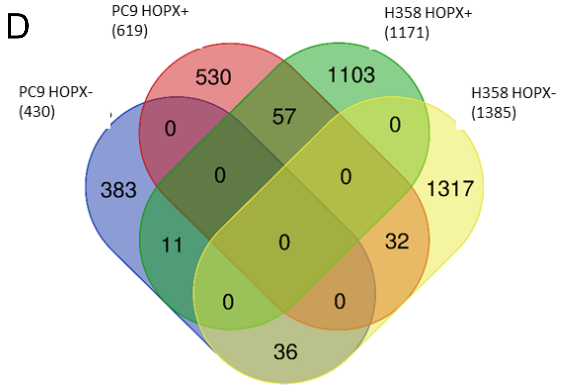

E

Comparison	Expected number	Observed number	Fold change	Significance
H358 HOPX+ vs. PC9 HOPX+	25	57	2.2	2.51E-8
H358 HOPX+ vs. PC9 HOPX-	17	11	0.6	0.05
H358 HOPX- vs. PC9 HOPX+	30	32	1.1	0.42
H358 HOPX- vs. PC9 HOPX-	21	36	1.7	1.56E-3

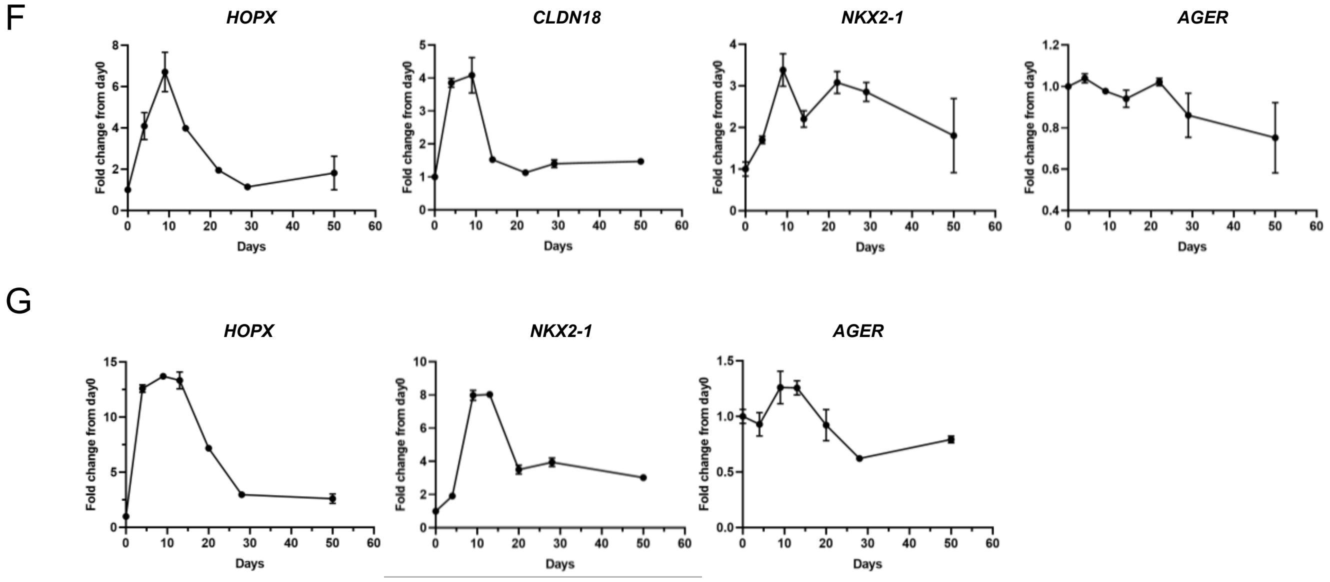

Figure S3. HOPX is specifically enriched in one DTP subpopulation and is transiently upregulated in DTP cells.

A). UMAP of scATAC-seq data showing accessibility at *HOPX, NKX2-1, AGER, SFTPC,* and *SFTPB* gene loci. The UMAP includes cell populations from both untreated-PC9 and DTP-PC9 cells as shown in Fig. 1C. **B)**. UMAP of scATAC-seq data showing accessibility at *HOPX, NKX2-1, AGER, SFTPC,* and *SFTPB* gene loci. The UMAP includes cell populations from both untreated-NCI-H358 and DTP-NCI-H358 cells as shown in Fig. 2C. **C)**. UMAP of scRNA-seq data showing mRNA level of *HOPX, NKX2-1* and *AGER*. The UMAP includes cell populations from both untreated-NCI-H358 and DTP-NCI-H358 cells as shown in Fig. 2F. **D)**. Venn gram showing signature genes overlapping between HOPX+ DTP subpopulation (cluster 6) and HOPX- DTP subpopulation (cluster 5) in PC9 model, as well as HOPX+ DTP subpopulation (cluster 3) and HOPX- DTP subpopulation (cluster 4) in NCI-H358 model. **E)**. Quantification of genes overlapping by hypergeometric analysis. **F).** mRNA levels of *HOPX, CLND18, NKX2-1,* and *AGER* at different time points (days 0, 4, 9, 14, 22, 29, 50) after PC9 cells were treated with 150nM Osimertinib. **G).** mRNA levels of *HOPX, NKX2-1,* and *AGER* at different time points (days 0, 4, 9, 13, 20, 28, 50) after NCI-H358 cells were treated with 500nM Sotorasib. Data are presented as means ± S.D. (n=3). Statistical significance was determined using Kruskal-Wallis-Test, $*p < 0.05$, $**p < 0.01$, $***p < 0.001$, $****p < 0.0001$.

Figure S4

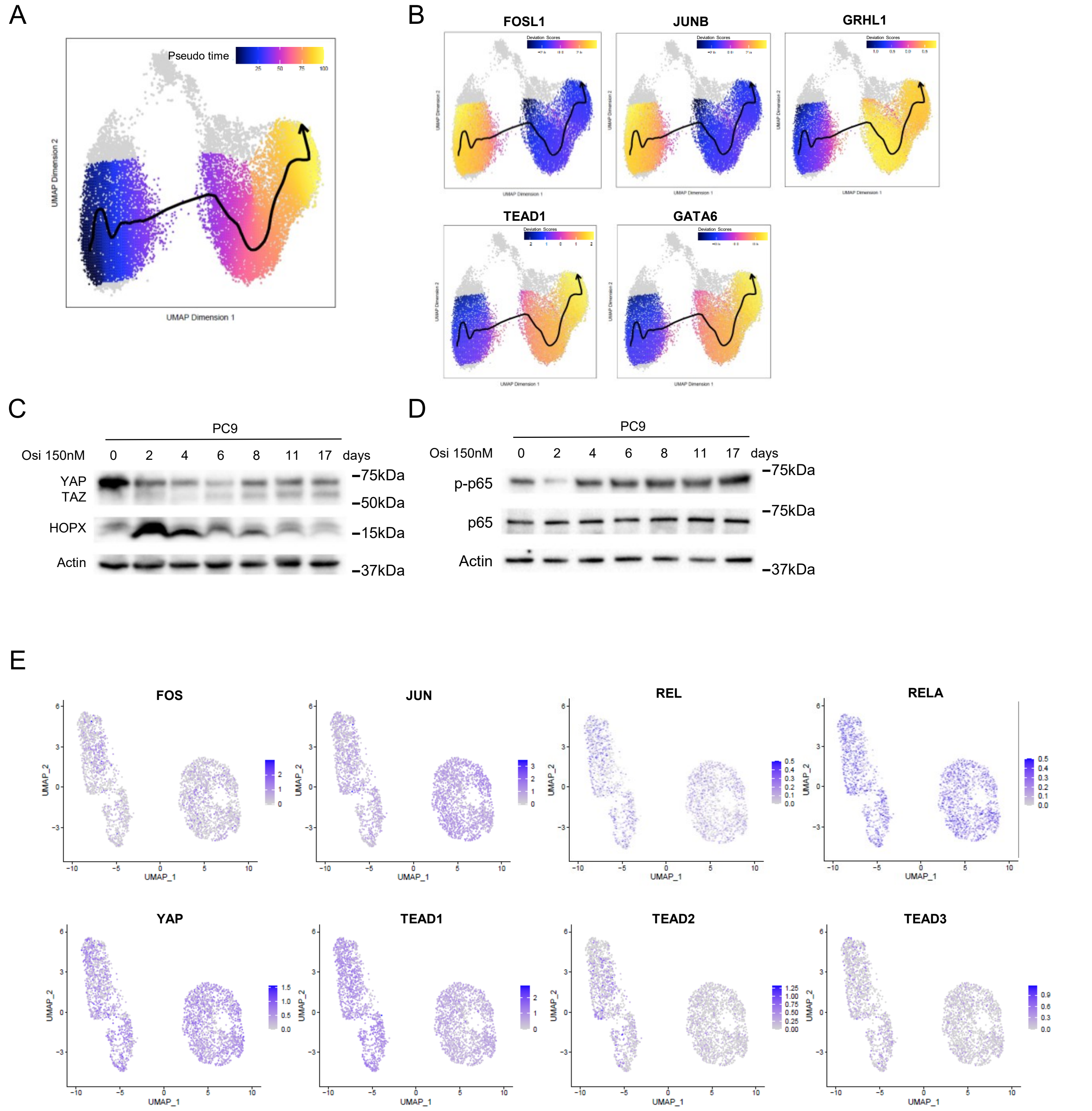
A
Pseudo time
25
50
75
100
UMAP Dimension 2
UMAP Dimension 1
B
FOSL1
JUNB
GRHL1
TEAD1
GATA6
Deviation Scores
C
PC9
Osi 150nM
0
2
4
6
8
11
17
days
YAP
TAZ
HOPX
Actin
75kDa
50kDa
15kDa
37kDa
D
PC9
Osi 150nM
0
2
4
6
8
11
17
days
p-p65
p65
Actin
75kDa
75kDa
37kDa
E
FOS
JUN
REL
RELA
YAP
TEAD1
TEAD2
TEAD3
UMAP_1
UMAP_2

Figure S4. HOPX regulation converging with NF-κB pathway during DTP development.

A). Pseudotime trajectory for the DTP evolution constructed by *ArchR* based on gene accessibility. Arrow represents directions of DTP cell state changes. **B).** Evolutionary trajectory from scATAC-seq and projected *FOLSL1, JUNB, GRHL1, TEADS1*, and *GATA6* gene accessibility on UMAP. **C).** YAP and TAZ levels measured by western blot on PC9 cells at 0, 2, 4, 6, 8, 11, and 17 days post initiation of 150nM Osimertinib treatment. The blots for HOPX, YAP, and Actin originate from the same set of independent experiments as in Figure 3E. Data are representative of three independent experiments. **D).** Phospho-p65 and p65 level measured by western blot on PC9 cells at 0, 2, 4, 6, 8, 11, and 17 days post initiation of 150nM Osimertinib treatment. **E)**. UMAP of scRNA-seq data showing mRNA level of *FOS, JUN, REL, RELA, YAP, TEAD1, TEAD2,* and *TEAD3*. The UMAP includes cell populations from both untreated-PC9 and DTP-PC9 cells as shown in Fig. S1A.

FigureS5

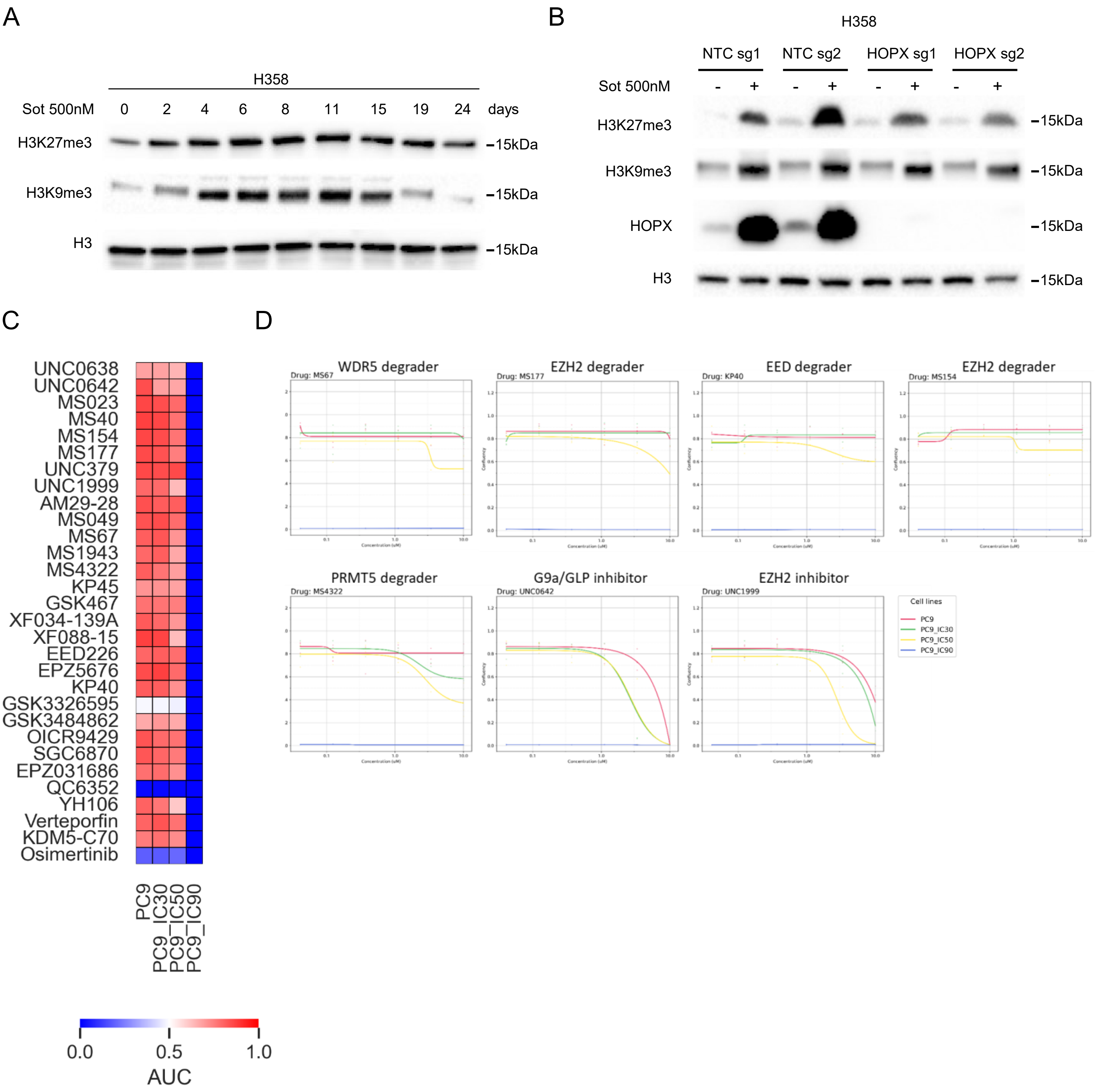
A
H358
Sot 500nM 0 2 4 6 8 11 15 19 24 days
H3K27me3
H3K9me3
H3
15kDa
B
H358
NTC sg1 NTC sg2 HOPX sg1 HOPX sg2
Sot 500nM - + - + - + - +
H3K27me3
H3K9me3
HOPX
H3
15kDa
C
UNC0638
UNC0642
MS023
MS40
MS154
MS177
UNC379
UNC1999
AM29-28
MS049
MS67
MS1943
MS4322
KP45
GSK467
XF034-139A
XF088-15
EED226
EPZ5676
KP40
GSK3326595
GSK3484862
OICR9429
SGC6870
EPZ031686
QC6352
YH106
Verteporfin
KDM5-C70
Osimertinib
PC9
PC9_IC30
PC9_IC50
PC9_IC90
0.0 0.5 1.0
AUC
D
WDR5 degrader
Drug: MS67
EZH2 degrader
Drug: MS177
EED degrader
Drug: KP40
EZH2 degrader
Drug: MS154
PRMT5 degrader
Drug: MS4322
G9a/GLP inhibitor
Drug: UNC0642
EZH2 inhibitor
Drug: UNC1999
Cell lines
PC9
PC9_IC30
PC9_IC50
PC9_IC90

Figure S5. The function of HOPX on DTP progression is mediated by maintaining the compressed chromatin structure at early DTP stage.

A). H3K27me3 and H3K9me3 levels measured by western blot on NCI-H358 cells at 0, 2, 4, 6, 8, 11, 15, 19, and 24 days post initiation of 500nM Sotorasib treatment. **B)**. Western blot for H3K27me3, H3K9me3 and HOPX levels in NCI-H358 cells transduced with non-target sgRNAs or HOPX sgRNAs with or without Sotorasib treatment. **C)**. Drug screen assay with a library containing 29 chemical compounds in combination with different Osimertinib dosage (IC30:2nM IC50:4nM, and IC90:3000nM). Heatmap showing AUC (area under the curve) of drug exposure dosage to cell confluency measured by imaging on PC9 cells after 9 days of treatment seeded at 100 cell per well. **D)**. Cell viability on untreated (red line) and Osimertinib treated (yellow and green) cells for 7 chemical compounds combined with Osimertinib treatment.

Table S1. qPCR primers, related to Key resources table

Name	Forward Primer	Reverse Primer
HOPX	TCAACAAGGTCGACAAGCAC	TCTGTGACGGATCTGCACTC
CLDN18	ACATGCTGGTGACTAACTTCTG	AAATGTGTACCTGGTCTGAACAG
NKX2-1	CAGGACACCATGAGGAACAGCG	GCCATGTTCTTGCTCACGTCCC
AGER	GTGTCCTTCCCAACGGCTC	ATTGCCTGGCACCGGAAAA
SETDB1	GCCTACAGCAAGGAACGTATCC	GTTGATGGCAGGCACACTTGGA
Suv39H2	CCATAAATGCTGGAGAAGAGCTG	GGTAACCTCTGCAAGTCACAGC
Suv39H1	CCGCCTACTATGGCAACATCTC	CTTGTGGCAAAGAAAGCGATGCG
EHMT2	GGTGAACAACCACCTGGAGGTA	AGGCTGACCATCTCCAAGTTCC
EHMT1	GCTTCAGAAGGTGCTCCTCATG	CTGAACCAGCATGTGGCAGATG
EZH1	CACCACATAGTCAGTGCTTCCTG	AGTCTGACAGCGAGAGTTAGCC
EZH2	GACCTCTGTCTTACTTGTGGAGC	CGTCAGATGGTGCCAGCAATAG
EED	GACGAGAACAGCAATCCAGACC	TCCTTCCAGGTGCATTTGGCGT
Suz12	CCATGCAGGAAATGGAAGAATGTC	CTGTCCAACGAAGAGTGAACTGC
Jarid2	GGACAAAGGCGTCCTCAATGAC	GCAGGCTCCTTGCTGAAACACA

Table S2. sgRNA sequences, related to Key resources table

	gRNA target sequence	oligo1	oligo2
HOPX gRNA 1	GCTCGCGGTCTCCGCCGACA	CACCGGCTCGCGG TCTCCGCCGACA	AAACTGTCGGCGG AGACCGCGAGCC
HOPX gRNA 2	GGCCTGCCTCGGCCGCGATG	CACCGGGCCTGCC TCGGCCGCGATG	AAACCATCGCGGCC GAGGCAGGCCC
non-target gRNA 1	ACGGAGGCTAAGCGTCGCAA	CACCGACGGAGGC TAAGCGTCGCAA	AAACTTGCGACGCT TAGCCTCCGTC
non-target gRNA 2	CGCTTCCGCGGCCCGTTCAA	CACCGCGCTTCCGC GGCCCGTTCAA	AAACTTGAACGGGC CGCGGAAGCGC

Table S4. Bayesian regulatory network node genes derived from signature genes of PC9 DTP clusters, related to Figure 1

Cluster5_sub-network_ one-layer node gene	Cluster6_sub-network1_ one-layer node gene	Cluster6_sub-network2_ one-layer node gene
ABHD14B	AFAP1	TMEM56
SPINK5	GJA5	SLC30A1
FANCD2	FBN1	CSNK1E
SMC2	SPARCL1	SEC24A
SFTA1P	CDH5	PPTC7
R3HDM1	EMCN	SBF1
MYL6B	COL18A1	NFKBIL1
CKAP5	CDH11	N4BP2
CENPF	AFAP1L1	ITPA
H2AFZ	LDB2	TAOK1
CDC20	PDZRN3	NFXL1
SULT1A1	PLEKHO2	CLIP1
SFTPC	IL10RA	C10orf18
DSN1	FSTL1	LOC151162
SNRPA1	PCOLCE	MAP3K2
MACROD2	SPON1	COMMD4
NAA15	LUM	C11orf48
CLDN18	COL8A1	IFT27
LAMP3	DCN	NOL12
RCCD1	CYTH4	CLOCK
STMN1	ROR2	SAPS2
RTKN2	LGI2	RPS19BP1
CDC7	TRMT61A	SGSM3
MRPL3	EMILIN1	PICK1
FAM162A	SIVA1	GGA1
C16orf75	RAB3IL1	CCDC12
CCDC58	TRIL	SCO2
AGER	PACS2	IGF2R
RFC2	NUDT14	TUBGCP6
MCM2	NDST1	ELFN2
CYB5A	OAF	CRELD2
GTSE1	MRC2	RHBDD3
NUF2	MTA1	MEGF6
DISP1	KIAA0427	TTLL3
SMC6	TEK	ZC3H6
TSPAN3	CACNA1C	KLF8
NUDT16P1	S1PR1	COL4A4
CHEK2	JAG2	COL4A3
CCNB2	CPXM1	C19orf60
CCNE2	DCHS1	TGFBRAP1
AURKB	PTPRB	KCNC3
FER1L4	COL6A1	MGAT5

USP31	C14orf79	CYP2R1
EPR1	PECAM1	CHKB-CPT1B
MRPL47	CLEC11A	KIAA1715
ANKRD26	BTBD6	ASXL2
NUDT16	BRF1	SDF2L1
MCM6	LHFP	COQ4
MAD2L1	FOXF1	TTLL1
KIF4A	MXRA7	ANKRD54
CCNA2	ZBTB42	TRABD
RFC4	APLNR	DNAL4
MSH6	COL6A3	SELO
TOPBP1	TIMP3	
CHEK1	COL6A2	
RANBP1	FIBIN	
GINS3	C1R	
CDCA5	COL5A1	
RNASE1	CCDC80	
CHAF1B	PDE3B	
GEN1	WAS	
NEK2	PDGFRA	
KNTC1	ERG	
	ANTXR1	
	PTRF	
	LILRB1	
	STAB1	
	BCAT2	
	TSPAN18	
	BMP6	
	PCDH17	
	KIAA1462	
	KDR	
	CYYR1	
	HEG1	
	CXorf36	
	CALHM2	
	SPRY1	
	FGD5	
	IGF1	
	ADAMTS9	
	PRSS23	
	HEPH	
	GPR124	
	CD93	
